# Combined PARP and WEE1 inhibition triggers anti-tumor immune response in BRCA1/2 wildtype triple-negative breast cancer

**DOI:** 10.1038/s41523-023-00568-5

**Published:** 2023-08-15

**Authors:** Zhi Ling Teo, Mark J. O’Connor, Stephanie Versaci, Kylie A. Clarke, Emmaline R. Brown, Luke W. Percy, Keilly Kuykhoven, Christopher P. Mintoff, Peter Savas, Balaji Virassamy, Stephen J. Luen, Ann Byrne, Sneha Sant, Geoffrey J. Lindeman, Phillip K. Darcy, Sherene Loi

**Affiliations:** 1 Peter MacCallum Cancer Centre, 305 Grattan Street, Melbourne, VIC 3000 Australia; 2 Sir Peter MacCallum Department of Oncology, The University of Melbourne, Parkville, Victoria 3010 Australia; 3 AstraZeneca Oncology, Cambridge, UK; 4 Cancer Biology and Stem Cells Division, The Walter and Eliza Hall Institute of Medical Research, Parkville, VIC Australia; 5 Department of Medicine, University of Melbourne, Parkville, VIC Australia; 6 Cancer Immunology Research, Peter MacCallum Cancer Centre, Melbourne, VIC Australia

**Keywords:** Cancer immunotherapy, Predictive markers

## Abstract

Novel therapeutic strategies that can effectively combine with immunotherapies are needed in the treatment of triple-negative breast cancer (TNBC). We demonstrate that combined PARP and WEE1 inhibition are synergistic in controlling tumour growth in *BRCA1/2* wild-type TNBC preclinical models. The PARP inhibitor (PARPi) olaparib combined with the WEE1 inhibitor (WEE1i) adavosertib triggered increases in anti-tumour immune responses, including STING pathway activation. Combinations with a STING agonist resulted in further improved durable tumour regression and significant improvements in survival outcomes in murine tumour models of *BRCA1/2* wild-type TNBC. In addition, we have identified baseline tumour-infiltrating lymphocyte (TIL) levels as a potential predictive biomarker of response to PARPi, WEE1i and immunotherapies in *BRCA1/2* wild-type TNBC.

## Introduction

Triple-negative breast cancer (TNBC) remains a highly aggressive breast cancer subtype with poor overall survival and a high probability of metastasis and death^1^. The clinical prognosis of relapsed TNBC is dismal, with a median survival of only 15 months.

Olaparib was the first poly(ADP-ribose) polymerase (PARP) inhibitor to be approved for the treatment of advanced breast cancers with *BRCA1/2-*inherited mutations and represents the first targeted-therapy approved for TNBC^2^. PARP is crucial for DNA repair. PARP inhibitors (PARPi) demonstrating clinical monotherapy activity result in PARP trapping onto the DNA, which during replication, results in replication fork stalling and collapse, leading to the formation of DNA double strand breaks (DSB)^3^. Cancer cells that are defective in homologous recombination repair such as those with *BRCA1/2* mutations, are unable to accurately repair these PARPi-induced DNA DSBs and undergo cell death^4^. The majority of *BRCA1/*2 mutant breast cancers are TNBC but only 10–15% of TNBC patients have inherited mutations in *BRCA1/2*^1^. Nonetheless, there are preclinical^5,6^ and clinical^7,8^ evidence for the activity of olaparib monotherapy in *BRCA1/2* wild-type cancers.

WEE1 protein kinase activity regulates progression through S-phase and the G2/M checkpoint by controlling CDK2 and CDK1 kinase activity, respectively^9,10^. WEE1 inhibition can abrogate G2 arrest, forcing cells with unrepaired DNA damage into mitosis and lead to mitotic catastrophe^11^ but also induce replication catastrophe in cancer cells undergoing high levels of replication stress^12,13^. As such, the combination of WEE1 inhibitors (WEE1i) with PARPi has the potential to increase anti-tumour activity over and above that of PARPi treatment alone by exacerbating PARPi-induced replication stress. Indeed, the PARPi/WEE1i combination has been demonstrated to be effective in small cell lung cancer, ovarian cancer PDX as well as preclinical breast cancer models regardless of *BRCA* mutation status^14–19^.

PARPi monotherapy has been shown to potently induce STING (stimulator of IFN genes)-mediated anti-tumour immune responses as well as activate the PD-1/PD-L1 immune checkpoint pathway in various cancer models^20–22^ including *BRCA-*mutant TNBC^23,24^. In these PARPi sensitive models, PARPi induces DNA damage, generating cytosolic double-stranded DNA which activates the STING pathway^20,21,23^. PARPi-induced increase in type I interferons via STING pathway activation^21^ as well as inhibition of GSK3β^24^ was shown to increase PD-L1 expression, contributing to PD-1/PD-L1 immune suppression which was reversed with the addition of PD-1/PD-L1 checkpoint blockade^20–22,24^. The addition of STING agonist has also been shown to improve therapeutic efficacy of PARP inhibition in *BRCA-*mutant TNBC^25^. Nonetheless, the effect of PARPi and/or WEE1i on the immune response in *BRCA* wild-type TNBC models remains undetermined. We hypothesise that combined PARP and WEE1 inhibition might likewise activate STING-dependent anti-tumour immune response in *BRCA* wild-type TNBC. In this study, we also sought to develop preclinical rationale for combining immunotherapies (namely anti-PD-1 and STING agonist) with PARP and WEE1 inhibition in *BRCA* wild-type TNBC. Increased lymphocytic infiltration has been repeatedly shown to be associated with improved prognosis and response to the mainstay therapies of TNBC including chemotherapy and immunotherapies^26–28^. However, results from these clinical trials show that the tumour microenvironment in the majority of TNBC patients lack pre-existing T cell infiltrates. Precision medicine has become an important aspect in the management of cancer patients. As such, we have sought to evaluate how levels of pre-existing T cell infiltrate might affect response to combined PARP and WEE1 inhibitors as well as if the combined treatment could stimulate a more immune-inflamed tumour microenvironment conducive for therapeutic response to immune checkpoint inhibitors.

Our findings demonstrate that combined treatment with olaparib (PARPi) and advertosertib (AZD1775; WEE1i) increased STING-mediated proinflammatory cytokine production in *BRCA1/2* wild-type TNBC models. Anti-tumour efficacy and immune responses were more prominent in tumours with higher levels of baseline T cell infiltrate. Our results have also identified baseline TIL levels as a potential predictive biomarker of PARPi, WEE1i and immunotherapy in *BRCA1/2* wild-type TNBC.

## Results

### Combined PARP and WEE1 inhibition interact synergistically to induce apoptosis, DNA damage and growth inhibition of *BRCA1/2* wild-type TNBC in vitro

**Fig. 1:**
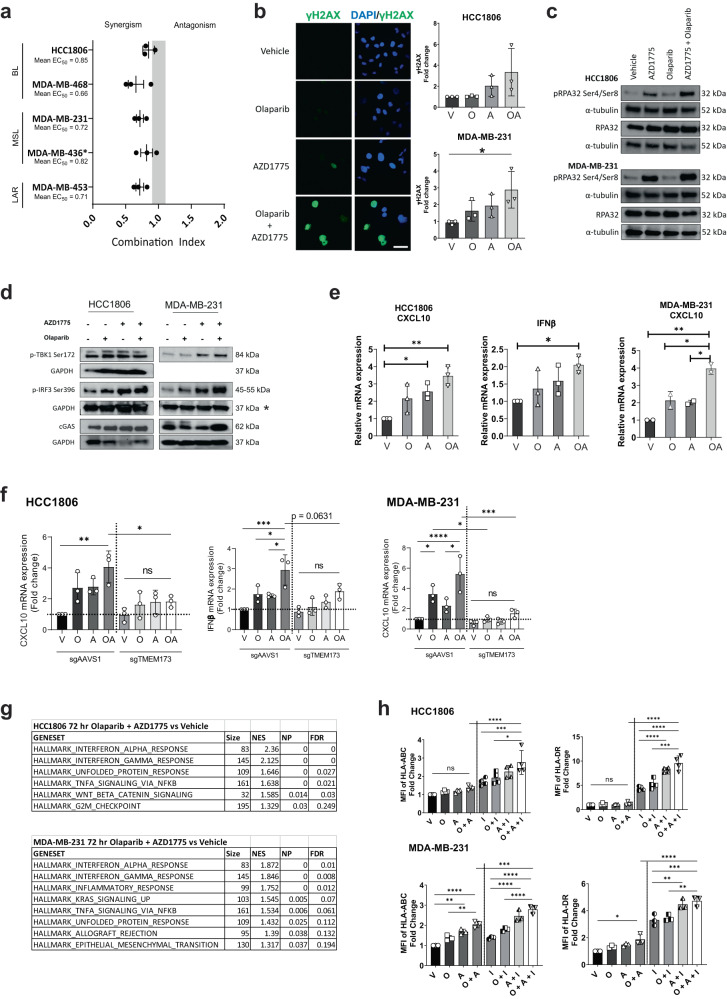
Combined PARP and WEE1 inhibitor treatment induces synergistic anti-tumour effects as well as STING-mediated inflammatory response in *BRCA1/2* wild-type TNBC. **a** Synergy quantitation via Chou-Talalay combination index (CI) values of olaparib and AZD1775 in TNBC cell lines. All cell lines are *BRCA1/2* wild type except MDA-MB-436 (*BRCA1* mutant indicated by *). Grey bar: CI values indicating additive effect. BL: Basal-like; MSL; Mesenchymal-like; LAR: Luminal androgen receptor. Data depicts mean ± s.d. **b** Images show immunofluorescence of γH2AX in MDA-MB-231 treated with DMSO (V), olaparib (O) and/or AZD1775 (A) for 72 h. Scale bar represents 50 µm. Graph shows mean fold change relative to vehicle controls ± s.d. analysed via flow cytometry. **c** Assessing protein expression of replication stress markers via Western blot after 24 h of indicated treatment. **d** Assessing protein expression of substrates in the STING pathway via Western blot after 24 (pIRF3 and pTBK1) and 72 (cGAS) hours of indicated treatment. Results in (**c**) and (**d**) are representative of at least 2 independent experiments. *: The GAPDH expression marked by * is the loading control for both pIRF3 Ser369 and pTBK1 Ser172 of the MDA-MB-231 cell line. **e** Gene expression via qRT-PCR in HCC1806 and MDA-MB-231 cells after 72 h treatment with olaparib and AZD1775. Data shows mean relative mRNA expression ± s.d. **f** Comparing gene expression levels in HCC1806 and MDA-MB-231 cells with STING knockout (sgTMEM173) vs sgAAVS1 control cells via qRT-PCR after 72 h treatment with olaparib and AZD1775. Data shows mean relative mRNA expression ± s.d. **g** Top ranked, upregulated GSEA Hallmarks in HCC1806 and MDA-MB-231 cells treated in vitro for 72 h with olaparib and AZD1775. Normalized *P* < 0.05, FDR < 0.25. **h** HCC1806 and MDA-MB-231 were treated for 72 h with vehicle, olaparib, AZD1775, and/or interferon (I; 5 ng/mL) and analysed via flow cytometry for tumour cell-surface expression of major histocompatibility complex (MHC) markers HLA-ABC and HLA-DR. Data depicts mean fold change of mean fluorescent intensity (MFI) relative to vehicle controls ± s.d. *P < 0.05; **P < 0.01; ***P < 0.001; ****P < 0.0001 by one-way ANOVA.

The effect of olaparib and AZD1775 on growth inhibition was quantitated in five human TNBC cell lines including four *BRCA1/2* wild-type and one *BRCA1* mutant cell lines (MDA-MB-436). Of the four *BRCA1/2* wild-type cell lines, MDA-MB-468 and HCC1806 are classified as basal-like, MDA-MB-231 is mesenchymal-like and MDA-MB-453 is of the luminal androgen receptor (LAR) subtype^29^. All five TNBC cell lines were similarly sensitive to AZD1775 with GI50 values ranged from 0.17 to 0.56 µM after 72 h treatment in vitro (Table 1). MDA-MB-231 and MDA-MB-453 were observed to be more resistant to olaparib monotherapy, both with GI50 values of >10 µM compared with the remaining cell lines with GI50 values ranging from 3.4–6.21 µM. The interaction between olaparib and AZD1775 was found to be synergistic in all five cell lines using the Chou and Talalay method of synergy quantitation^30^ (Fig. 1a). We further validated the synergistic interaction of the drug combination in three *BRCA1/2* wild-type TNBC cell lines using a clonogenic assay (Supplementary Fig. 1a). These findings suggest that AZD1775 can sensitise *BRCA1/2* wild-type TNBC cell lines to olaparib monotherapy.

**Table 1:** GI50 values of olaparib and AZD1775 in TNBC cell lines.

Cell lines	*BRCA1/2* mutation status	GI50 of olaparib (µM)	GI50 of AZD1775 (µM)
HCC1806	Wild type	3.40	0.26
MDA-MB-468	Wild type	6.21	0.19
MDA-MB-231	Wild type	>10	0.21
MDA-MB-436	*BRCA1* mutation	4.80	0.56
MDA-MB-453	Wild type	>10	0.17

Combined inhibition of PARP and WEE1 in vitro led to significant increase in apoptosis as well as DNA damage as indicated by the increased phosphorylation levels of Ser139 on histone 2AX (γH2AX) compared with the vehicle treatment groups (Supplementary Fig. 1b and Fig. 1b). PARP and WEE1 inhibitors have been previously shown to induce replication stress as single-agents via PARP trapping and nucleotide depletion, respectively^31,32^. We confirmed this through increased phosphorylation of replication protein A 32 kDa subunit (RPA32) with the combination treatment compared with vehicle and single-agent controls (Fig. 1c). These results suggest that olaparib and AZD1775 in combination induces synergistic anti-tumour effects in *BRCA1/2* wild-type TNBC through the increase of apoptosis, DNA damage and replication stress.

### Combined inhibitor treatment induces STING-mediated inflammatory response and upregulates MHC I expression on tumour cells

It has been shown that PARP inhibitors increase STING-mediated inflammatory responses in several tumour types^20–23^. In breast cancer, the increase in STING pathway activation was observed to be pronounced in *BRCA1/2* mutant but not in *BRCA1/2* wild-type tumours^23^. Cytosolic cyclic GMP-AMP synthase (cGAS) senses pathogenic or self-DNA and activates STING signalling. Levels of cGAS as well as phosphorylation of STING pathway effectors TANK-binding kinase 1 (TBK1) and IFN regulatory factor 3 (IRF3) on Ser172 and Ser196, respectively^32,33^, were assessed to determine if STING pathway is activated in response to olaparib and/or AZD1775 treatment. Consistent with previous reports^23^, our results show that there was little to no increase in phosphorylation of pTBK1 and pIRF3 with olaparib single-agent treatment in the *BRCA1/2* wild-type HCC1806 and MDA-MB-231 cells (Fig. 1d). Addition of AZD1775 augmented the levels of cGAS in MDA-MB-231 as well as phosphorylation of pIRF3 in both cell lines when combined with olaparib which suggests an increase in cGAS/STING pathway activation. IFNβ and CXCL10 are proinflammatory cytokine and chemokine known to be primarily induced by STING-dependent signalling^33,34^. Similar to pTBK1 and pIRF3, we show that the mRNA levels of IFNβ and CXCL10 were not different in olaparib single-agent treated cells but were significantly increased in the combined inhibitor treated cells compared with the vehicle controls (Fig. 1e). *IFNB1* was not detectable in the MDA-MB-231 cells consistent with previous reports^35,36^ and the addition of PARP and/or WEE1 inhibitors did not increase *IFNB1* levels above detection levels for this cell line. We show that the increase in *IFNB1* in HCC1806 and *CXCL10* expression in both cell lines in response to single-agent and combined inhibitor treatment is dependent on STING. CRISPR-mediated knockout of *STING* (sgTMEM173) inhibited the increase in *CXCL10* and *IFNB1* expression in response to olaparib and AZD1775 which was otherwise observed in the sgAAVS1 control cells (Fig. 1f). These results indicate that the increase in *CXCL10* and *IFNB1* in response to combined PARP and WEE1 inhibition is dependent on the STING pathway.

Consistent with the activation of the STING pathway, gene set enrichment analysis (GSEA) of 3′RNAseq data of both HCC1806 and MDA-MB-231 cell lines show that both single-agent treatment of olaparib and AZD1775 in both cell lines induced a significant increase in the Hallmarks “Interferon Alpha Response” and “Interferon Gamma Response” (Fig. 1g). These Hallmark gene sets had the highest normalised enrichment score for both cell lines. Despite the upregulation of these inflammatory response hallmarks observed with single-agent treatments (Supplementary Fig. 1c), significant increase in gene expression of interferon stimulated genes (ISGs) was only observed in response to combined inhibitor treatment (Supplementary Fig. 1d). Specifically, we observed significant upregulation of ISGs in the combined inhibitor treated cells including *CXCL10, DDX58, IFIT3, ISG15, MX1, OAS2, OASL* and *TNF* (Supplementary Fig. 1e). We also observed upregulated expression of genes encoding major histocompatibility complex (MHC) class I and II molecules (*HLA-A, B, G, DMA*) as well as genes encoding peptide transporter (*Tap1*), transporter-MHC interactions (*TAPBP*) and processor of MHC class I T cell epitopes (*PSMB9*) in the combined inhibitor treatment groups suggesting that combined PARP and WEE1 inhibition can increase tumour antigen presentation capabilities of TNBC (Supplementary Fig. 1f). We show a corresponding significant increase in tumour cell surface expression of MHC class I molecules (HLA-ABC and HLA-DR) in response to combined inhibitor treatment in MDA-MB-231, however, this was not observed in HCC1806 (Fig. 1h). We also treated these cells with interferon-γ (IFNγ) to simulate a tumour microenvironment with an active immune response. We show that the effects of combined PARP and WEE1 inhibitor treatment in both cell lines are potentiated in the presence of IFNγ which resulted in the significant increase expression of the MHC class I molecules compared to cells treated with combined PARP and WEE1 inhibitors and cells treated with only IFNγ alone. Loss of MHC expression is one of the immune escape strategies employed by malignant cells to avoid T cell recognition^37^. Our results suggest that combined PARP and WEE1 inhibition can enhance antigen presentation capability of the *BRCA1/2* wild-type TNBC cells particularly in the presence of inflammatory cytokines.

Our observation of increased DNA damage (Fig. 1b) and enrichment of unfolded protein response from the GSEA analysis (Fig. 1g) suggests potential stress-induced release of damage-associated molecular patterns (DAMPs) in response to combined inhibitor treatment. We therefore assessed the expression of cell-surface calreticulin expression on several TNBC cell lines and show that its expression is upregulated following combined inhibitor treatment compared with vehicle (Supplementary Fig. 1g). The exposure of cell surface calreticulin suggests the induction of immunogenic cell death which may promote anti-tumour immune responses^38^. A summary of the key findings in the human cell lines are listed in Supplementary Table 1. Overall, these results suggest that combined olaparib and AZD1775 treatment increases tumour cell immunogenicity that may trigger an anti-tumour response.

### Combined olaparib and AZD1775 enhances anti-tumour immunity of a primary TNBC tumour model in vivo

**Fig. 2:**
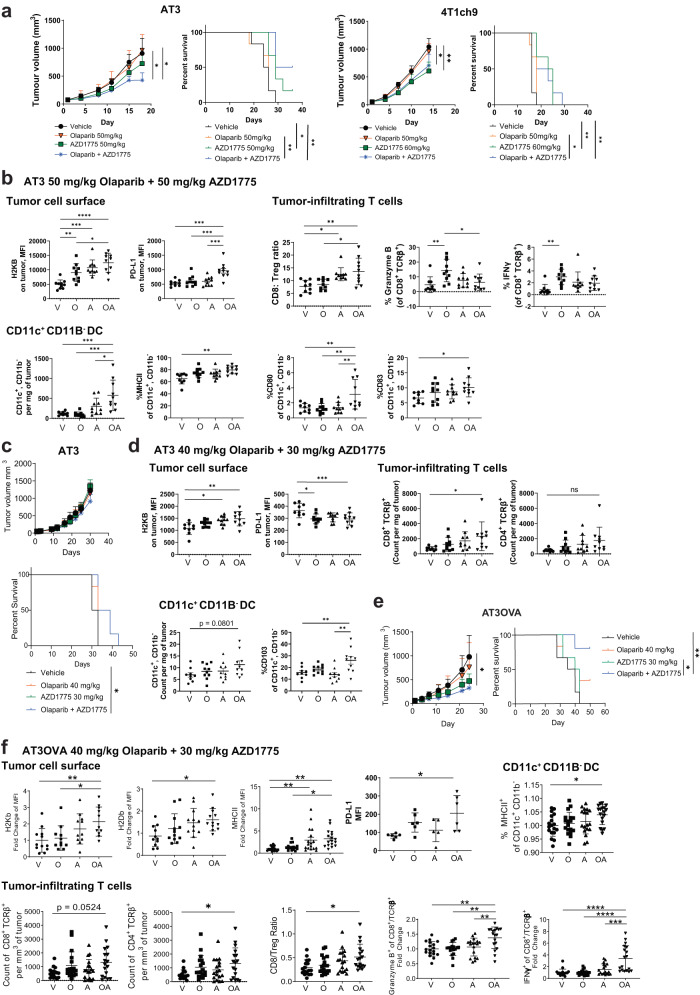
Combined treatment with olaparib and AZD1775 increases anti-tumour immunity in vivo. **a**, **c**, **e** Growth and survival curves of AT3, 4T1ch9 and AT3OVA tumours. Tumour-bearing mice were treated with vehicle, olaparib and/or AZD1775. 6 mice per treatment group. Data shows mean tumour volume ± s.d. **b**, **d** AT3 tumour-bearing mice were treated with vehicle, olaparib and/or AZD1775 for 15 days. Tumours and were harvested and analysed via flow cytometry. 10 mice per treatment group. Data shows mean ± s.d. **f** AT3OVA tumour-bearing mice were treated with vehicle, olaparib and/or AZD1775 for 16 days. PD-L1 expression on AT3OVA tumours was acquired at 8 days of treatment. All other markers were acquired at 16 days of treatment. 6 mice per treatment group. Data pooled from 3 independent experiments. Data shows mean ± s.d. ns: non-significant. *P < 0.05; **P < 0.01; ***P < 0.001; ****P < 0.0001 by one-way ANOVA for FACS analyses, two-way ANOVA for tumour growth curves and log-rank (Mantel-Cox) test for survival curves.

We next examined the efficacy of combined olaparib and AZD1775 treatment in syngeneic *BRCA1/2* wild-type AT3 and 4T1ch9 TNBC models in immune-competent mice. We show that AZD1775 but not olaparib monotherapy improved survival in both AT3 and 4T1ch9 models, suggesting that these two *BRCA1/2* wild-type models of TNBC are both relatively sensitive to AZD1775 but are resistant to olaparib. Combined PARP and WEE1 inhibitor treatment at clinically relevant doses reduced the growth rates of both tumour models as well as significantly improved survival compared with olaparib monotherapy and the vehicle treated controls (Fig. 2a). Nonetheless, it is apparent that the combined inhibitor treatment does not lead to tumour regressions and all mice in the treatment group were eventually sacrificed due to tumour burden reaching the ethical limit.

Our observation that olaparib and AZD1775 directly enhances MHCI expression in vitro led us to examine the tumour immune microenvironment in response to the treatment combination in vivo. Using the AT3 tumour model, we show that the combined inhibitor treatment upregulated tumour cell surface expression of MHCI (H2KB) (Fig. 2b) which validates our in vitro findings. Immune checkpoint PD-L1 (programmed cell death-ligand 1) expression was also significantly upregulated in AT3 tumours from mice treated with the combination of olaparib and AZD1775 compared with those from the monotherapy and vehicle treatment groups which is suggestive of the presence of immune suppression via the PD-1/PD-L1 pathway despite the immunogenic microenvironment induced by the combination treatment. We also observed a significant decrease in CD80 and CD86 T cell costimulatory molecules (Supplementary Fig. 2a).

Dendritic cells (DCs) are central regulators of the adaptive immune response, in part by transporting and cross-presenting tumour antigens to activate cytotoxic T lymphocytes. STING pathway activation has been shown to mediate maturation of DCs^39^. Consistent with this, we show that the combination treatment also significantly increased the number of intra-tumoral CD11c^+^ CD11b^−^ DCs in vivo compared to the monotherapy and vehicle treatment groups (Fig. 2b). Combined PARP and WEE1 inhibitor treatment also significantly increased the proportion of DCs expressing MHC class II, and costimulatory molecules CD80 and CD83 which is indicative of DC maturation, antigen presentation ability as well as ability to induce T cell receptor signalling and promote T cell activation. In line with this, we also observed increases, albeit not significant, in numbers of CD8^+^ TCRβ^+^ and CD4^+^ TCRβ^+^ cells in response to combined olaparib and AZD1775 treatment compared with vehicle controls (Supplementary Fig. 2a). Combined inhibitor treatment also significantly increased CD8/Treg ratio compared with olaparib monotherapy and vehicle control groups (Fig. 2b) suggesting that the combined inhibitor treatment can mediate a shift in the tumour microenvironment to one that promotes an anti-tumour immune response. Olaparib monotherapy was shown to significantly increase the proportion of granzyme B and IFNγ produced by CD8^+^ T cells compared to vehicle treated controls, reflecting an increase in functional activity and cytotoxic potential of these T cells. However, this increase was not observed in the combined inhibitor treatment group (Fig. 2b).

The 4T1ch9 model is a highly aggressive metastatic TNBC *BRCA1/2* wild-type tumour model that metastasizes to the liver, lungs, bone and brain^40^ and animals have been shown to succumb to lethal metastatic disease by 6 weeks post tumour implantation. The 4T1 models are also known to induce production of CD11b^+^Gr1^+^ myeloid suppressor cells^41^ which generates a highly immune suppressed tumour immune microenvironment, which is typical of patients with metastatic breast cancer. We show that 4T1ch9 tumour immunogenicity and tumour immune microenvironment remained unaltered in response to combined PARP and WEE1 inhibitor treatment (Supplementary Fig. 2b) suggesting that the anti-tumour efficacy in the 4T1ch9 metastatic model as seen in Fig. 2a might be largely independent of the immune system.

### Reduced doses of olaparib and AZD1775: impact on anti-tumour efficacy and immunity

We show that clinically relevant doses of 50 mg/kg olaparib in combination with 50–60 mg/kg AZD1775 is well-tolerated in both AT3 and 4T1ch9 models on the basis of minimal changes in the weights of the mice (Supplementary Fig. 2c). Nonetheless, overlapping toxicity profiles have been reported for PARPi, WEE1i, anti-PD-1 and STING agonist, in particular, fatigue as well as gastrointestinal toxicities such as nausea and diarrhoea^42–45^. Dose reductions and/or sequential administration of these inhibitors would be necessary to result in reduced toxicity so that the combination might be better tolerated in patients^14,46^. Therefore, it is of interest to understand how dose reductions of olaparib and AZD1775 might affect the anti-tumour efficacy as well as immune responses in our tumour models to facilitate translation into the clinics. We show that a reduced dose of olaparib (50 mg/kg to 40 mg/kg) and AZD1775 (50 mg/kg to 30 mg/kg) in combination is similarly well-tolerated in the AT3 model compared with the higher doses (Supplementary Fig. 2c). While the combined inhibitor treatment still significantly improved survival compared with vehicle treated controls, it is clear from the growth curves that the degree of growth inhibition with the reduced doses in the AT3 model is diminished compared with the higher doses in the same model (Fig. 2c). Nonetheless, AZD1775 at 30 mg/kg in both monotherapy and combined treatment groups resulted in a decrease in phosphorylation of CDC2 (CDK1) at Tyr15 indicating effective inhibition of WEE1 in the AT3 tumours at this reduced dose (Supplementary Fig. 2d). However, there was no appreciable change in phosphorylation of CHK1 at Ser345 in the AZD1775 monotherapy and combination treatment groups compared with the vehicle treatment groups in the AT3 tumours (lower band indicated by the arrow in Supplementary Fig. 2d).

Combined inhibitor treatment at reduced doses showed a similar significant increase in MHCI expression on cell surface of AT3 tumours (Fig. 2d) similar to that observed with the higher doses. However, expression of PD-L1 was significantly reduced on cells in the combined treatment group at lower doses compared with vehicle treated controls, contrary to the significant increase observed in the higher doses possibly due to the lack of DNA damage induced with the lower doses in cells treated with single-agent AZD1775 or the combination as inferred by the phosphorylation levels of CHK1 at Ser345.

At higher doses, expression of T cell costimulatory molecules CD80 and CD86 were significantly downregulated. In contrast, CD80 on AT3 was significantly upregulated but that of CD86 remained unchanged in response to lower doses of combined inhibitor treatment (Supplementary Fig. 2e). At both high and low doses, we also observed similar increases in the number of CD11c^+^ CD11b^-^ DC per mg of tumour as well as in proportion the of CD11c^+^ CD11b^-^ DC expressing CD103. Similar trends were observed for the number of CD8 TCRβ^+^and CD4 TCRβ^+^ cells as well as proportion of granzyme B and IFNγ positive CD8^+^ T cells across the treatment groups (Fig. 2d and Supplementary Fig. 2e).

In summary, the results suggest that the anti-tumour efficacy of combined inhibitor treatment and the corresponding immune responses are dose-dependent in the AT3 tumour model. Despite diminished efficacy, the combined inhibitor treatment at the reduced doses nevertheless resulted in increases in anti-tumour immune responses which we hypothesise could be potentiated with further immunotherapy combinations.

### Higher levels of pre-existing tumour-infiltrating T cells are associated with augmented anti-tumour efficacy and immune response

The quantity of pre-existing tumour-infiltrating lymphocytes (TILs) observed at diagnosis has been shown to be prognostic for early-stage TNBC patients treated with chemotherapy^26^. It was therefore of relevance to determine if and how differences in quantity of pre-existing TILs might affect the anti-tumour efficacy and immune response of combined PARP and WEE1 inhibition in the *BRCA1/2* wild-type TNBC models. We show that the AT3OVA TNBC tumour model has greater number of pre-existing T cells per mg of tumour due to the presence of the immunogenic OVA antigen on the cell surface compared with the AT3 and the 4T1ch9 tumours (Supplementary Fig. 3a).

In vitro treatment of the AT3OVA cells with olaparib and AZD1775 showed an increase in cGAS/STING pathway activation (Supplementary Fig. 3b). We show that the reduced doses of olaparib and AZD1775 induced a similar level of anti-tumour efficacy in the AT3OVA model compared with the AT3 model treated at a higher dose (Figs. 2e and 2a, respectively). With these reduced doses, we observed on-target inhibition of WEE1 indicated by a decrease in phosphorylation of CDC2 at Tyr15 as well as increase in DNA damage signalling as indicated by the phosphorylation of CHK1 at Ser345 in the AZD1775 monotherapy and combination treatment groups compared with the vehicle treatment groups of the AT3OVA model, similar to that of the AT3 tumours treated with higher doses (Supplementary Fig. 3c).

The anti-tumour immune responses in the AT3OVA tumours of combined inhibitor treatment at reduced doses was also observed to be greater compared to the AT3 model of both doses (Fig. 2b, d, f). MHCI (H2KB and H2DB), MHCII, CD80, CD86 and PD-L1 expression on the AT3OVA tumours were all significantly increased in response to combined inhibitor treatment compared with vehicle controls (Fig. 2f and Supplementary Fig. 3d). Significant increases in number of CD4^+^ T cells and CD8:Treg ratio were also observed. The proportion of mature DCs (MHCII^+^ expressing CD11c^+^ CD11b^-^ DCs) were significantly increased in the combined inhibitor treatment group compared with vehicle treatment group (Fig. 2f). The proportion of KI67 and PD-1 expressing CD4^+^ T cells was significantly increased in the combined inhibitor treatment group which is indicative of increased proliferation and potential engagement of the immune suppressive PD-1/PD-L1 checkpoint pathway in line with the significant increase of PD-L1 expression on the tumour (Supplementary Fig. 3d; Fig. 2f). The proportions of granzyme B and IFNγ produced by CD8^+^ and CD4^+^ T cells were significantly increased in the combined inhibitor treatment group compared to the monotherapy and vehicle treatment groups (Fig. 2f and Supplementary Fig. 3d), suggesting an increase in T cell activation and cytotoxicity.

Having a moderate level of pre-existing TILs in the AT3OVA model was observed to provide a conducive tumour microenvironment that synergised well with olaparib and AZD1775 despite the reduced doses to induce a potent anti-tumour immune response.

### Anti-tumour efficacy of combined olaparib and AZD1775 is dependent on CD8^+^ and CD4^+^ T cells

**Fig. 3:**
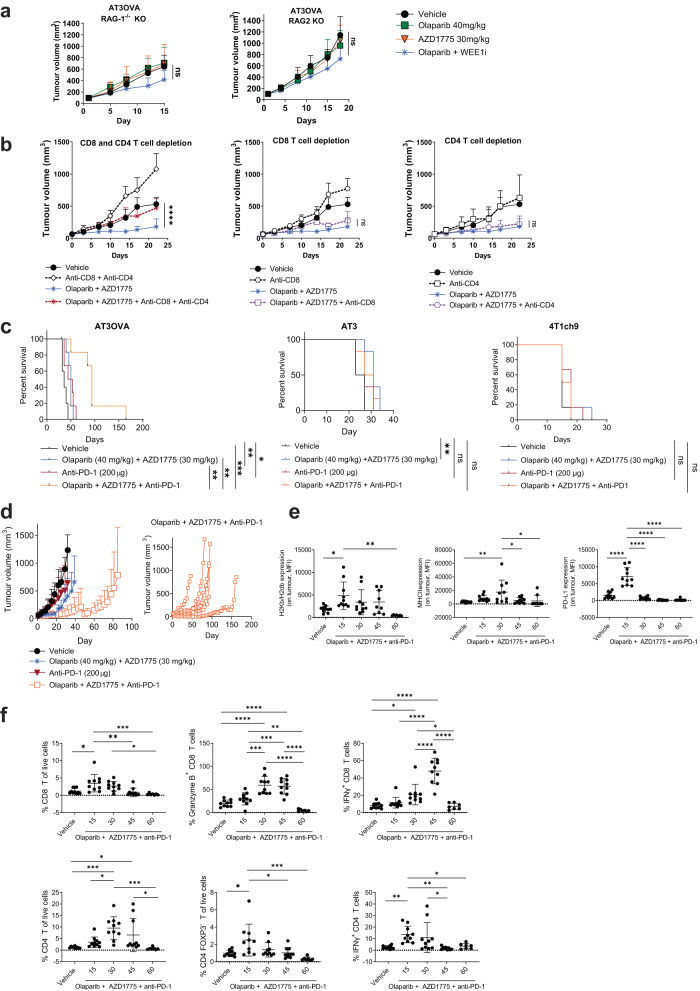
Combination of checkpoint blockade with DDR inhibitor treatment confers benefit to tumours with moderate levels of pre-existing T cells, however, treatment resistance emerges via immune editing. **a** AT3OVA cells were injected into RAG-1^-/-^ and RAG-2^-/-^γc^-/-^ mice and treated with vehicle, olaparib and/or AZD1775. Data shows mean tumour volume ± s.d. **b** AT3OVA tumour-bearing mice were treated with vehicle, olaparib and AZD1775 with or without CD8 (YTS 169.4; 250 µg) and CD4 (GK1.5; 250 µg) T cell depleting antibodies. The depletion antibodies were administered on day 0 (the day before targeted therapy treatment), day 1 (the first day of targeted-therapy treatment), day 7, 14 and 21. LTF-2 (Rat IgG2b) was administered as an isotype control for the depletion antibodies. Data shows mean tumour volume ± s.d. **c** Survival curves of AT3OVA, AT3 and 4T1ch9 tumour-bearing mice (C57Bl/6 and BALB/c wild-type strains) treated with olaparib, AZD1775 and/or anti-PD-1 (clone: RMP1-14). 6 mice per treatment group. **d** Corresponding tumour growth curves of AT3OVA tumour-bearing mice described in (**c**). Graph on the right shows the tumour growth curves of individual mice treated with olaparib, AZD1775 and anti-PD-1. **e**, **f** AT3OVA tumour-bearing mice were treated with olaparib and AZD1775 5 days/week for 15, 30 45 or 60 days. 10 mice per treatment group. Tumours were harvested analysed via flow cytometry. Data shows mean ± s.d. ns: non-significant. *P < 0.05; **P < 0.01; ***P < 0.001; ****P < 0.0001 by one-way ANOVA for FACS analyses, two-way ANOVA for tumour growth curves and log-rank (Mantel-Cox) test for survival curves.

The functional contribution made by host lymphocytes during treatment of olaparib and AZD1775 in the AT3OVA model was further investigated in C57BL/6 strains which lack mature B cells and T cells (RAG-1^-/-^ mice) or mature B cells, T cells and natural killer (NK) cells (RAG-2^-/-^ γc^-/-^ mice). In these two immune-deficient strains, combined treatment of olaparib and AZD1775 conferred no significant difference in AT3OVA tumour growth compared with vehicle or single-agent treatment groups (Fig. 3a). The similarity in response to combined inhibitor treatment in RAG-1^-/-^ and RAG-2^-/-^ γc^-/-^ mice suggests that NK cells contribute little to the anti-tumour activity of the treatment. Given our observation of increased in number and activity of tumour-infiltrating CD8^+^ and CD4^+^ T cells in response to combined inhibitor treatment, we reasoned that the T cells might be central to the anti-tumour efficacy of combined inhibitor treatment. To confirm this, we treated immune-competent mice (wild type C57BL/6 strain) with anti-CD8 and anti-CD4 antibodies to deplete CD8^+^ and CD4^+^ T cells, respectively, in addition to them receiving olaparib and AZD1775 treatment. The depletion of these immune subsets was verified via flow cytometry (Supplementary Fig. 4a). We show that the tumour growth control exerted with combined olaparib and AD1775 treatment was abrogated when both CD8^+^ and CD4^+^ T cells were depleted (Fig. 3b). Whereas depletion of either CD8^+^ or CD4^+^ T cells alone did not affect treatment efficacy of olaparib and AZD1775 suggesting that the cytotoxicity of both CD8^+^ and CD4^+^ T cells is essential for the anti-tumour efficacy of combined PARP and WEE1 inhibition in the *BRCA1/2* wild-type AT3OVA TNBC model.

### Combination of immune checkpoint blockade with DDR inhibitor treatment confers benefit to tumours with moderate levels of pre-existing T cells

PARP and WEE1 inhibitors in combination is not sufficient to eradicate *BRCA1/2* wild-type TNBC tumours regardless of whether they have low (AT3, 4T1ch9) or moderate (AT3OVA) levels of pre-exsiting TILs. Nonetheless, combined PARP and WEE1 inhibitor treatment was shown to promote anti-tumour immunity in the AT3 and AT3OVA tumour microenvironment, potentially rendering the tumours more sensitive to immunotherapy.

The crosstalk between PARP inhibition and activation of the PD-L1/PD-1 immune checkpoint axis has previously been demonstrated^20,24^ and have prompted the exploration of combining PARP and immune checkpoint inhibition in clinical trials^47,48^ with the aim of understanding and overcoming PARP inhibitor resistance as well as immune suppression via PD-1/PD-L1. In the TOPACIO trial^48^, the combination of niraparib and pembrolizumab showed promising efficacy but this was mostly limited to those with *BRCA-*mutated tumours. We sought to determine whether PD-1 blockade would potentiate the anti-tumour activity of combined PARP and WEE1 inhibition. We evaluated this combination in the AT3OVA model and show that the combination of reduced doses olaparib, AZD1775 and anti-PD-1 significantly improved survival and tumour growth control compared to the combined olaparib and AZD1775 treatment group (*p* < 0.001; Fig. 3c). The median duration of survival for the mice treated with the three-drug combination was 93.5 days compared with 43 days in the anti-PD-1 group and 50 days in the olaparib and AZD1775 combined treatment group. Tumour regression was observed in 1/6 mice in the three-drug combination group and complete tumour clearance was observed for approximately 30 days before tumour started progressing at day 60 and reached ethical limit at day 162. The results suggest that immune suppression via the PD-1/PD-L1 axis is activated by combined olaparib and AZD1775 treatment in the *BRCA1/2* wild-type AT3OVA model which was overcome, albeit briefly, with further incorporation of immune checkpoint blockade in the treatment regimen.

Consistent with the anti-tumour effect of combined PARP and WEE1 inhibitors being dependent on T cells, the anti-tumour immune response to olaparib and AZD1775 treatment was not as pronounced in the AT3 compared with the AT3OVA tumours. Given the downregulation of PD-L1 on the AT3 tumours, it was unsurprising that the addition of anti-PD-1 conferred no further benefit in the AT3 as well as the 4T1ch9 models (Fig. 3c). The lower doses of olaparib and AZD1775 were effective in decreasing the phosphorylation levels of CDC2 at Tyr15 in the 4T1ch9 tumours (Supplementary Fig. 4b). We also observed a corresponding increase in phosphorylation of CHK1 at Ser 345 in the 4T1ch9 tumours, in contrast to the results observed in AT3 tumours treated with reduced doses, suggesting that the lack of anti-tumour response observed in these two models treated with combined olaparib, AZD1775 and anti-PD-1 was not associated with the level of DNA damage signalling in the tumours indicated by the phosphorylation of CHK1.

Despite inducing a significant increase in CD8^+^ T cell infiltration in the tumour, the combined olaparib and AZD1775 treatment at lower doses led to a significant reduction of PD-L1 expression on the AT3 tumours (Fig. 2d) which could explain the striking lack of response to anti-PD-1 treatment. In contrast, reduced doses of olaparib and AZD1775 was observed to significantly increase PD-L1 expression on AT3OVA tumours at day 8 of treatment as well as at day 16, albeit not significant (Supplementary Fig. 4c). These results suggest that the difference in PD-L1 expression between the two models could be influenced by the quantity of pre-existing TILs in the tumours.

We have shown that higher doses of olaparib and AZD1775 significantly increased PD-L1 expression on AT3 tumours (Fig. 2b) and this corresponded with a significant improvement in anti-tumour efficacy of the three-drug combination compared with the olaparib and AZD1775 combination treatment group but not when compared with anti-PD-1 single-agent treatment group (Supplementary Fig. 4d). The three-drug combination at these increased doses were observed to be well-tolerated (Supplementary Fig. 4e). Nonetheless, these results suggest that the combination of olaparib and AZD1775, regardless of dose, does not sensitise tumours with low levels of pre-existing TILs to anti-PD-1 treatment.

For tumours with moderate levels of baseline TILs, PD-1 checkpoint blockade confers additional anti-tumour activity in combination with the olaparib and WEE1 inhibition, even at reduced doses, to achieve more durable anti-tumour efficacy.

### Emergence of treatment resistance to PD-1 checkpoint blockade via immune editing

While the combination of anti-PD-1 and DDR inhibitor treatment extended survival of AT3OVA mice, treatment resistance eventually emerged (Fig. 3d). To gain potential mechanistic insight for the observed treatment resistance over time, AT3OVA tumour-bearing mice were treated with vehicle for 15 days or the combination of olaparib, AZD1775 and anti-PD-1 for 15, 30, 45 and 60 days. Tumours were then harvested and analysed at these time points. Flow cytometry analysis of tumour cell surface markers and tumour-filtrated immune cell subsets over time revealed an overall phenomenon of immune evasion.

The three-drug combination was shown to be immunostimulatory. On day 15 of treatment, we observed a significant upregulation of tumour immunogenicity markers (MHCI and PD-L1), significant increase in the proportion of tumour-infiltrating CD8^+^ and CD4^+^/FOXP3^-^ T effector cells as well as the production of IFNγ by CD4^+^ T cells (Fig. 3e, f). The MHCII expression on tumour cells as well as the production of granzyme B by CD8^+^ T cells peaked around day 30 whereas IFNγ produced by CD8^+^ T cells continued to increase until day 45. All aforementioned markers were observed to be downregulated to similar levels observed in the vehicle treated groups by day 60 which corresponds to the observed emergence of tumour progression (Fig. 3d). Strikingly, PD-L1 expression on the tumours markedly decreased by day 30, which is suggestive of a potential mechanism of acquired resistance to PD-1 blockade^49,50^ in this model.

### Combination with STING agonist improves durability of response of olaparib and AZD1775 in TNBC models with low levels of pre-existing TILs

We have shown that the combination of PARP and WEE1 inhibitors induce an increase in immune responses as well as an increase in STING pathway activation. Based on these findings, we hypothesized that pharmacological activation of the STING pathway using a STING agonist could enhance the anti-tumour activity of PARP and WEE1 inhibitors. STING agonists have also been shown to be well-suited for combination with immune checkpoint blockade, particularly anti-PD-1, to overcome resistance to PD-1 blockade as well as render poorly immunogenic tumours to become sensitive to immune checkpoint inhibitors^51–58^. We reasoned that the addition of a STING agonist could also be used to sensitise tumours with low levels of pre-existing T cells to PD-1 blockade.

**Fig. 4:**
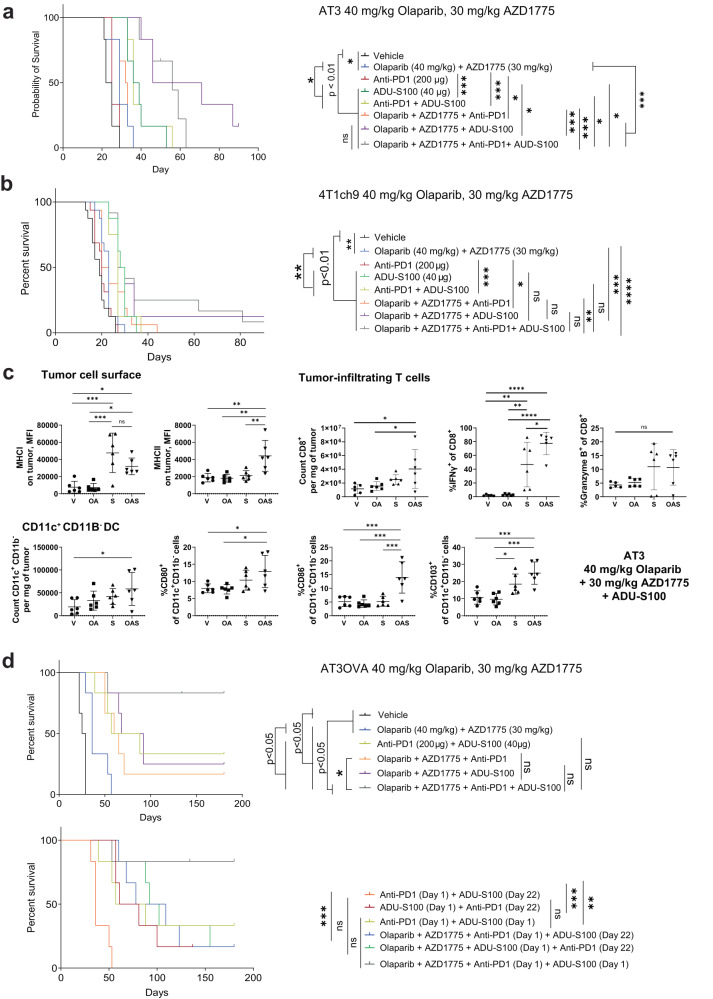
Resistance to olaparib and AZD1775 can be overcome with the addition of STING agonist. **a**, **b**, **d** AT3, 4T1ch9 and AT3OVA tumour-bearing mice were treated with the indicated compounds. AT3 and AT3OVA tumour models: 6 mice per treatment group. 4T1ch9 tumour model: 8 mice per treatment group. Data pooled from 2 independent experiments. Data shows mean ± s.d. **c** AT3 tumour-bearing mice were treated with vehicle, olaparib and/or AZD1775 for 15 days. Tumours and were harvested and analysed via flow cytometry. 6 mice per treatment group. Data shows mean ± s.d. ns: non-significant. *P < 0.05; **P < 0.01; ***P < 0.001; ****P < 0.0001 by log-rank (Mantel-Cox) test for survival curves and one-way ANOVA.

We treated the AT3 and 4T1ch9 tumour models with olaparib, AZD1775, ADU-S100 (STING agonist) and/or a PD-1 inhibitor. STING agonist as monotherapy was observed to have potent anti-tumour efficacy in both models, with significant improvement to survival compared to vehicle, as well as combined olaparib and AZD1775 treatment groups (Fig. 4a, b). However, contrary to our hypothesis, the combination of STING agonist and anti-PD-1 did not confer any additional survival benefit to STING agonist monotherapy in these models that have low levels of pre-existing TILs. Notably, the addition of STING agonist to olaparib and AZD1775 performed significantly better in terms of overall survival compared with STING agonist monotherapy, with tumour regression in 2/6 and complete tumour clearance of 1/6 of the AT3 tumours (>50 days), whereas no tumour regression or tumour clearance was observed for ADU-S100 monotherapy group (Supplementary Fig. 5a). We show that the three-drug combination of olaparib, AZD1775 and ADU-S100 significantly increased expression of MHCI and MHCII on AT3 tumour cell surface compared with vehicle controls (Fig. 4c). There was no significant difference in the expression of CD80, CD86 or PD-L1 on AT3 tumour cells in the group treated with the three-drug combination compared with the vehicle treated group (Supplementary Fig. 5b). We further show that this three-drug combination significantly increased the number of tumour-infiltrating CD8^+^ T cells. Strikingly, the production of IFNγ by CD8^+^ T cells was significantly greater in the three-drug combination treatment group compared with all other groups. We also observed a non-significant increase in the production of granzyme B by CD8^+^ T cells. The three-drug combination treatment also concurrently induced significant increases in the number of intra-tumoral CD11c^+^ CD11b^-^ DCs as well as the proportion of DCs expressing CD103 and costimulatory molecules CD80 and CD86. These results demonstrate that the marked anti-tumour efficacy observed upon combining olaparib, AZD1775 and STING agonist is a result of increased tumour immunogenicity, tumour infiltration and activation of DCs, as well as tumour infiltration and interferon signalling of CD8 T cells.

In the 4T1ch9 model, there was no significant difference in survival between the three-drug combination (olaparib, AZD1775 and ADU-S100), and ADU-S100 monotherapy treatment groups (Fig. 4b), perhaps unsurprising as the combination of olaparib and AZD1775 did not elicit anti-tumor immune responses in this model (Supplementary Fig. 2b). Nonetheless, the combination of olaparib, AZD1775 and ADU-S100 resulted in complete tumour clearance of 1/6 of the 4T1ch9 tumours (>50 days; Supplementary Fig. 5c). Similar to the AT3 model, no tumour regression or tumour clearance was observed for ADU-S100 monotherapy in the 4T1ch9 model. There was also no significant difference in survival outcomes between the four-combination compared with the three-drug combination of olaparib, AZD1775 and ADU-S100 in the 4T1ch9 model suggesting that the addition of anti-PD-1 confers no additional survival benefit to the combination of combination of olaparib, AZD1775 and ADU-S100.

To summarise these results, the three-drug combination of STING agonist, with reduced doses of olaparib and AZD1775 can confer significant survival advantage in the TNBC tumour models with low level of pre-existing T cell infiltrate compared with the combination of olaparib and AZD1775. However, the survival benefits of this three-drug combination might be limited in patients with metastatic disease where the tumors are typically in highly immune suppressive tumor microenvironments.

We observed that higher doses of olaparib (50 mg/kg) and AZD1775 (50 mg/kg) significantly increased PD-L1 expression on AT3 tumours (Fig. 2b) which corresponded with a limited but significant improvement in anti-tumour efficacy of olaparib, AZD1775 and anti-PD-1 (Supplementary Fig. 4d) compared with the two-drug combination of olaparib and AZD1775. We wanted to determine if the increase in PD-L1 expression due to treatment with higher doses of olaparib and AZD1775 would affect the efficacy of the four-drug combination in this model. Complete tumour clearance in 1/6 mice was observed in the four-drug combination, however, no difference in overall survival was observed when comparing the four-drug combination with the three-drug combination of olaparib, AZD1775 and ADU-S100 (Supplementary Fig. 6a, b), suggesting that there is no survival benefit to increasing the doses of olaparib and AZD1775 in the four-drug combination in tumours with low levels of pre-existing TILs. The three-drug and four-drug combinations with varying doses of olaparib and AZD1775 were observed to be tolerable across the tumour models (Supplementary Fig. 6c, d).

### Combination with STING agonist and anti-PD-1 achieves complete tumour clearance in TNBC models with moderate levels of pre-existing TILs

We next assessed the efficacy of combining anti-PD-1 and STING agonist with reduced doses of olaparib and AZD1775 in the AT3OVA model which has moderate levels of pre-existing TILs. We show that complete tumour regression was achieved with the four-drug combination in this model and that this treatment combination significantly outperforms all other treatment groups in terms of tumour growth control and survival with 5 out of 6 (66%) mice remaining tumour-free for more than 100 days (Top panel of Fig. 4d, and Supplementary Fig. 7a). A summary of the key findings from the animal studies are listed in Supplementary Table 2.

To facilitate translation into the clinic, we also assessed the efficacy of concurrent compared with sequenced administration of the immunotherapies in this model. For concurrent treatment regimen, AT3OVA tumour-bearing mice were treated with all 4 drugs on the same day (Day 1). For sequential administration regimens, the tumour-bearing mice were either treated with olaparib, AZD1775 and anti-PD-1 or olaparib, AZD1775 and ADU-S100 on day 1, followed by ADU-S100 or anti-PD-1 on day 22, respectively. We show that the concurrent administration of the four-drug combination still conferred the greatest survival benefit (Fig. 4d and Supplementary Fig. 7a). The concurrent administration of four-drug combination treatment was also well-tolerated in this model (Supplementary Fig. 7b).

Taken together, these results demonstrate that the therapeutic efficacy of PARP and WEE1 inhibitors can be further potentiated with the addition of STING agonist and anti-PD-1 to induce complete tumour clearance and improve survival outcomes in *BRCA1/2* wild-type TNBC with moderate levels of pre-existing TILs.

## Discussion

The paucity of pre-existing TILs presages poor clinical prognosis as well as responsiveness to the standard of care treatments for TNBC including chemotherapy and immune checkpoint inhibitors^59^. As such, it has become fundamentally important to understand the immune response to new treatment regimens to optimize treatment strategies that can effectively promote a proinflammatory tumour microenvironment and improve clinical response rates.

In this study, we show that WEE1 inhibition can sensitise *BRCA1/2* wild-type TNBC to PARP inhibitors resulting in synergistic anti-tumour efficacy with increased DNA damage, apoptosis, replication stress, and STING pathway activation. Concurrent therapy with PARPi and WEE1i at doses mimicking those used in combination in clinical trials^60^ was shown to trigger proinflammatory, anti-tumour immune responses in *BRCA1/2* wild-type AT3OVA and AT3 tumours. We also show that the anti-tumour efficacy of olaparib and AZD1775 was dependent on both CD8 and CD4 T cells. Unsurprisingly, having a higher baseline of TILs (AT3OVA vs AT3) was associated with greater levels of T cell infiltrates as well as T cell activation and cytotoxicity in response to combined olaparib and AZD1775 treatment. This corresponded with the increased expression of PD-L1 as well as CD80 and CD86 molecules on the AT3OVA tumours. While PD-L1 was similarly upregulated, the AT3 tumours were still able to engage immune evasive strategies by downregulating CD80 and CD86 molecules. CD28 is a costimulatory molecule essential for T cell activation which binds to CD80 (B7-1) and CD86 (B7-2) molecules. The presence of CD28, and its co-stimulation by CD80 and CD86 was shown to be critical for the rescue of exhausted T cells after PD-1 blockade^61^. Consistent with this, we show that olaparib and AZD1775 sensitized the AT3OVA but not the AT3 tumours to anti-PD-1 treatment. These results highlight the potential significance of evaluating positive costimulatory molecules in addition to the expression of inhibitory receptors on exhausted CD8 T cells to improve identification of patients who would derive most benefit from immune checkpoint inhibitors.

STING agonists have the ability to elicit both innate and adaptive immune responses, producing inflammatory cytokines that can remodel the tumour microenvironment^56^ which make them good candidates for non-inflamed tumours characterised by the lack of pre-existing TILs. Indeed, we show that STING agonism synergised with PARP and WEE1 inhibition which markedly increased proinflammatory anti-tumour immunity in the AT3 tumours resulting in tumour regression and prolonged survival. Our results suggest that the anti-tumour benefits of this three-drug combination might be limited in the metastatic setting which would require further studies involving additional tumour models. Nonetheless, given that this scenario is typical in patients with advanced TNBC, this combination should be further evaluated in the clinic. Interestingly, the three-drug combination (olaparib, AZD1775 and STING agonist) did not elicit PD-1/PD-L1-mediated immune suppression. Correspondingly, the addition of anti-PD-1 to the three-drug combination did not provide further survival benefit. In contrast, we demonstrate that concurrent treatment with STING agonist, anti-PD-1, olaparib and AZD1775 in AT3OVA tumours can overcome anti-PD-1 treatment resistance to result in durable tumour clearance. Our findings reiterate the clinical relevance of baseline TILs as a potential parameter in predicting response to PARP, WEE1 inhibitors and immunotherapies in *BRCA1/2* wild-type TNBC. Further work in additional mouse models and patient cohorts would be needed to define the threshold of percentage TILs at baseline to aid translation into clinics.

Clinically relevant as well as reduced doses of olaparib and AZD1775 in were evaluated in this study to facilitate translation of PARPi, WEE1i and immunotherapy treatment combinations into the clinics. We demonstrated proof-of-principle that the three- and four-drug treatment regimens were well-tolerated as well as efficacious with lower doses of olaparib and AZD1775. The combination of therapeutic agents tend to be the more efficacious approach compared to monotherapy as it targets multiple essential cancer cell-sustaining pathways which also contributes to the mitigation of drug resistance^57^. Nonetheless, there are inherent challenges of combining molecularly targeted agents^58^ due to overlapping toxicities and/or unexpected interaction between the drugs mechanisms of action. Nanoparticles for drug delivery^62^ hold significant potential as a means to address these issues by coordinating the pharmacokinetics and tissue exposure profiles of each agent to enable a more predictable translation of safety and efficacy profiles of drug compounds into the clinic.

Together, these findings further our translational understanding of therapeutics targeting the DNA damage response pathways in the context of *BRCA1/2* wild-type TNBC and how their therapeutic efficacy may be potentiated immunotherapies.

## Methods

### Cell lines

The human cell lines were obtained from ATCC (In Vitro Technologies). They have been authenticated by short tandem repeat analysis and were maintained in complete DMEM or RPMI1640 (Gibco). AT3^63^ and AT3OVA mouse TNBC cell lines were kindly provided by Professor Phillip Darcy (Peter MacCallum Cancer Centre, VIC, Australia) and maintained in complete SAFC DMEM. The 4T1ch9^64^ mouse TNBC cell line was kindly provided by Professor Robin Anderson (Olivia Newton-John Cancer Research Institute, VIC, Australia) and maintained in RPMI1640. All culture media were supplemented with 10% heat-inactivated foetal bovine serum. We have previously characterised all 3 mouse models and have shown that they were wild type for *Brca1* and *Brca2*^65^. All human and mouse cell lines have been verified to be negative for mycoplasma contamination and were maintained at 37 °C in a 5% CO_2_ incubator.

### CRISPR/Cas9-mediated knockout of *STING* (TMEM173)

Single guide RNA (sgRNA) sequences CATTACAACAACCTGCTACG and TGAAAAAGGGAATTTCAACG targeting human STING were delivered via nucleofection into HCC1806 and MDA-MB-231 cells. sgRNA sequence CGGGGACACAGGAUCCCUGG targeting human AAVS1 were used as negative controls. The Cas9 nuclease and sgRNAs were assembled at a ratio of 1:4 prior to nucleofection. The Cas9/sgRNA ribonucleotide (RNP) complex was incubated for 10 min at room temperature. 0.4 × 10^6^ cells were lifted and washed with PBS then resuspended in SE nucleofector solution. The RNP complex and cell suspension were transferred into Nucleocuvette vessels and pulse code CH125 was used for both cell lines. Knockdown was confirmed by western blot analysis.

### Drug compounds

Olaparib (AZD2281; PARP inhibitor) and AZD1775 (WEE1 inhibitor) were supplied by AstraZeneca via a Materials Transfer Agreement. ADU-S100 (STING agonist) was purchased from Chemietek. The dose of olaparib used for in vitro assays for all cell lines except MDA-MB-231 was 1 µM unless otherwise indicated. MDA-MB-231 was treated with 5 µM olaparib unless otherwise indicated. The dose of AZD1775 used for in vitro assays for all cell lines was 200 nM unless otherwise indicated. All drugs for in vitro use were reconstituted in DMSO. AZD1775 and olaparib were reconstituted in 0.5% methylcellulose and 10% 2 hydroxypropyl β-cyclodextrin, respectively (Sigma-Aldrich), for in vivo treatments. Immune checkpoint and depletion antibodies as well as their corresponding isotype controls used in in vivo experiments were obtained from BioXCell: anti-mouse PD-1 mAb (clone: RMP1-14, BE0146), anti-mouse CD8 mAb (clone: YTS 169.4, BE0117), anti-mouse CD4 mAb (clone: GK1.5, BE0003-1), Rat IgG2a isotype control mAb (clone:2A3, BE0089) and Rat IgG2b isotype control mAb (clone: LTF2, BE0090).

### Viability assay

To obtain GI50 values for each drug, viability of cells was assessed across a range of doses to an inhibitor. Cells were plated in 96-well white-walled plates and treated with escalating doses of the inhibitor. After 72 h, cell viability was determined based on quantitation of ATP present using CellTitre-Glo® Assay (Promega) luminescence read using Cytation™ 5 (BioTek). Curve fitting was performed using GraphPad Prism software. At least 3 independent experiments were performed per drug for each cell line.

### Combination treatment synergy quantitation

Drug combination studies were performed according to the Chou-Talalay method of synergy quantitation^30^. MDA-MB-436, MDA-MB-468, HCC1806, MDA-MB-231, and MDA-MB-453 cells were treated in vitro for 72 h with the combination of olaparib and AZD1775 over a range of concentrations held at a fixed ratio based on the GI50 (drug concentration required for 50% cell growth inhibition) of each drug specific for each cell line. CalcuSyn 2.0 (Biosoft) was used to determine the combination index (CI) which offers quantitative definition for additive effect (CI = 1), synergism (CI < 1), and antagonism (CI > 1) of drug combinations. At least 3 independent experiments per cell line were performed.

### Clonogenic assay

Clonogenic assays were conducted in 6-well plates where cells were exposed to vehicle or indicated treatments continuously for 2 weeks to allow for colony growth. HCC1806 cells were treated with 1 µM of olaparib and 50 nM of AZD1775, MDA-MB-231 cells were treated with 5 µM olaparib and 200 nM AZD1775, and MDA-MB-468 were treated with 0.5 µM of olaparib and 250 nM of AZD1775. Treatments were replaced every 3–4 days. Cells were fixed with 10% trichloroacetic acid and stained with 0.4% sulforhodamine B. At least 3 independent experiments per cell line were performed.

### Apoptosis analysis

Cells were plated in 24-well plates and treated the following day with the indicated agents for 72 h. Cells were then resuspended in 100 µL 1x annexin V binding buffer (5x: 50 mM HEPES, 700 mM NaCl, 12.5 mM CaCl_2_, pH7.4) and stained with 1 µg/ml propidium iodide and APC-conjugated Annexin V (1:100 dilution, 405717, Becton Dickinson). Cells were analysed using BD Biosciences LSR II flow cytometer. All experiments were performed in triplicate with three independent experiments. The gating strategy is provided in Supplementary Fig. 8a.

### Flow cytometry analysis of γH2AX

Cells were seeded in 24-well plates and treated the following day with the indicated agents. Cells were stained with a viability marker (LIVE/DEAD™ Fixable Yellow Dead cell stain kit, ThermoFisher Scientific) before resuspended in 4% paraformaldehyde (Sigma-Aldrich). Cells were permeabilised by resuspending in 90% methanol. Cells were then stained with p-γH2AX Ser139 (1:50 dilution. 9718, Cell Signalling Technology) overnight at 4 °C. The next day, cells were incubated with fluorochrome-conjugated secondary antibody Anti-rabbit IgG (H + L), F(ab')2 Fragment - Alexa Fluor® 488 Conjugate (1:200 dilution, 4408, Cell Signalling Technology). Flow cytometry analysis was performed on an LSR II flow cytometer (BD Biosciences). FACS data was analysed using FlowJo version 10 software (Tree Star Inc., USA). All experiments were performed in triplicate with at least three independent experiments. The gating strategy is provided in Supplementary Fig. 8b.

### Immunofluorescence analysis of γ-H2AX

Cells were seeded into ibidi 8-well µ-Slide (Ibidi, Germany) in media. After 24 h, media was removed and cells received their treatment. After treatment cells were washed and fixed in 4% paraformaldehyde. Cells were then blocked and permeabilised in blocking buffer (1X PBS + 5% BSA + 0.3% Triton X-100). Cells were incubated in primary phospho-histone γ-H2AX (Ser139) rabbit mAb (1:200 dilution in blocking buffer; 9718, Cell Signaling Technology) overnight at 4 °C in the dark. Cells were then washed before being incubated in the secondary antibody Anti-rabbit IgG (H + L), F(ab')2 Fragment - Alexa Fluor® 488 Conjugate (4408, Cell Signaling Technology); 1:1000 dilution in blocking buffer. After washing cells were counterstained with DAPI (0.5 µg/ml) for 5 minutes. Images were acquired using an Olympus Fluoview FV3000 confocal microscope system using a ×60 objective. All experiments were performed in triplicate with at least three independent experiments.

### Assessing MHC expression on tumour cell surface

Cells were seeded in 24-well plates and treated the next day with indicated treatments. After 72 h, the adherent cells were lifted using Tryple (Gibco) and stained with HLA-ABC (clone G46-2.6, 1:200 dilution, 555553, BD Biosciences), HLA-DR (clone LT43, 1:200 dilution, 502515, BD Biosciences) and a viability marker (1:400 dilution, LIVE/DEAD™ Fixable Yellow Dead cell stain kit, ThermoFisher Scientific) before resuspended in 4% paraformaldehyde (Sigma-Aldrich). Cells were analysed using the BD LSR II cell analyser. FACS data was analysed using FlowJo version 10 software (Tree Star Inc., USA). All experiments were performed in triplicate with three independent experiments. The gating strategy is provided in Supplementary Fig. 9a.

### Assessing calreticulin expression on tumour cell surface

Cells were seeded in 24-well plates and treated the next day with indicated treatments. After 72 h, cells in suspension and adherent cells were collected and centrifuged at 1400 rpm at 4 °C for 4 min. Cells were washed once with PBS and stained with Alexa Fluor®647-conjugated calreticulin antibody (1:50 dilution, clone: EPR3924, ab196159 Abcam). After 30 min incubation on ice, cells were washed with PBS and resuspended in 10 µg/mL propidium iodide. After incubation for 30 min at room temperature, cells were analysed using the BD LSR II cell analyser. FACS data was analysed using FlowJo version 10 software (Tree Star Inc., USA). All experiments were performed in triplicate with three independent experiments. The gating strategy is provided in Supplementary Fig. 9b.

### Western blot

Cells (2.5 × 10^5^/well) were seeded/well in 6-well tissue culture plates and allowed to adhere overnight. Cells were treated the next day as indicated. Cultured cells were lysed in RIPA buffer (1 mM EDTA pH8.0, 1% w/v NP-40, 0.05% w/v Na deoxycholate, 0.1% w/v SDS, 50 mM NaF, 1 mM pyrophosphate) supplemented with cOmplete™ mini protease inhibitor cocktail tablet (Roche) and PhosSTOP™ phosphatase inhibitor tablet (Roche). Lysates were centrifuged at 13,000 rpm × 10 min at 4 °C. Protein concentrations were calculated based on DC™ protein assay (Biorad) generated standard curve. Protein lysates (5 to 10 µg /lane) were run on mini-PROTEAN® TGX precast gels (Biorad), using Precision Plus Protein Dual Colour Standards (1610374, BioRad) for molecular weight estimation. The protein samples were then transferred to PVDF membrane (Trans-Blot Turbo RTA Midi 0.2 μm PVDF Transfer Kit, 1704273, BioRad) using the Trans-Blot Turbo Transfer System (Biorad). Membranes were blocked in 5% skim milk powder (for antibodies from Bethyl laboratories) or 5% BSA (for antibodies from Cell Signalling Technologies) for 1 h. One membrane (Fig. 1d and Supplementary Fig. 10) was cut horizontally at the 75 kDa mark, guided by the protein standards that were run on the first and last lanes of that gel. The top half of the membrane was used to probe for pTBK1 Ser172 (84 kDa) and the bottom half for pIRF3 Ser396 (45–55 kDa; bottom) separately. All membranes were incubated with the following primary antibodies from Cell signalling technologies: p-CHK1 Ser345 (1:1000 dilution, 2348), p-TBK1 Ser172 (1:1000 dilution, 5483), p-IRF3 Ser396 (1:1000 dilution, 4947), STING (1:1000 dilution, 13647), GAPDH (1:3000 dilution, 5174), α-tubulin (1:1000 dilution, 2144), and β-actin (1:1000 dilution, 4970). The following antibodies from Bethyl Laboratories Inc were used: RPA32 (1:3000 dilution, A300-244A) and p-RPA32 Ser4/Ser8 (1:2000 dilution, A300-245A). The membranes were washed 3 times for 10 minutes in 0.1% TBS-T and then incubated for an hour at room temperature with goat anti-rabbit Ig HRP-linked secondary antibody from CST (1:3000 dilution for GAPDH, RPA32 and pRPA32 Ser4/8, 1:2000 dilution for β-actin and α-tubulin, 1:1000 for the rest, 7074), detected using Pierce™ ECL Western Blotting Substrate (32106, Thermo Fisher Scientific) and captured via X-ray film or ChemiDoc^™^ MP Imaging System (BioRad). Samples in each presented immunoblot were derived from the same experiment. Unprocessed immunoblot images are in Supplementary Figs. 10 and 11.

### 3′ RNA sequencing

MDA-MB-231 and HCC1806 cells were seeded into 6-well plates and treated the following day as indicated for 72 h. Total cell RNA from 3 independent experiments was extracted using PureLink RNA Mini Kit (ThermoFisher Scientific) with on-column DNAse treatment according to manufacturer’s instructions. The quality and integrity of the total RNA extracted was determined using TapeStation 2200 system (Agilent Technologies, VIC) and Qubit RNA High Sensitivity assay kit (ThermoFisher Scientific). 500 ng total RNA was used for library preparation according to manufacturer’s instructions (QuantSeq 3' mRNA-Seq FWD, Lexogen). Indexed libraries were pooled and sequenced on a NextSeq500 (Illumina). Briefly, the library was generated with an oligo-dT containing the Illumina Read2 linker and a random forward primer containing the Illumina Read1 linker. The library was then amplified with PCR primers containing sample indices and the Illumina clustering sequences. Five to fifteen million single-end 75 bp reads were generated per sample.

### GSEA analysis

Gene Set Enrichment Analysis (GSEA) was performed using the GSEA tool on the entire normalised RNA expression count matrix without limiting the input to only differentially expressed genes. The Hallmark collection of 50 pre-defined gene sets and C5 Gene Ontology (GO) collection of pre-defined gene sets from the Molecular Signatures Database (MSigDB, Broad Institute) were used for analysis. The gene sets included in the analysis were limited to those that contained between 15 and 500 genes. Permutation was conducted 1000 times according to default weighted enrichment statistics and using difference of class metrics to calculate and rank genes according to their differential expression levels between two treatment groups. Significant gene sets were those defined with a nominal *p* < 0.05. Calculation of the false discovery rate (FDR) was used to correct for multiple comparisons and gene set sizes. Significantly enriched gene sets with FDR < 0.25 were selected for hypothesis generation.

### qRT-PCR

1 µg of total RNA/sample was then used to synthesise cDNA using the SuperScript™ VILO™ cDNA Synthesis Kit with ezDNase removal of genomic DNA following the manufacturer’s instructions (including no RT controls for each sample). PCR amplification of cDNA (diluted 1:5 in nuclease-free dH_2_O) was conducted using TaqMan Gene Expression Assays (Thermostat Scientific) for 18S (endogenous control; Hs03003631_g1), CXCL10 (Hs00171042_m1), IFNB1 (Hs02621180_s1), IFNγ (Hs00989291_m1), DDX58 (Hs01061436_m1), IFIT3 (Hs01922752_s1), ISG15 (Hs01921425_s1), OASL (Hs00984387_m1), TNF (Hs01113624_g1), MX1 (Hs00895608_m1), and OAS2 (Hs00942643_m1). Reactions were carried out in triplicate with TaqMan Fast Advanced Master mix in Microamp optical 96-well plates (Applied Biosystems). Reactions were run on the StepOne Plus Real Time PCR machine (Applied Biosystem) following the protocol: 50 °C 2 min (1 cycle), 95 °C 20 s (1 cycle) followed by 40 cycles of 95 °C 1 s, 60 °C 20 s. StepOne Plus software analysis was used to calculate comparative Ct (ΔΔCt) (relative quantitation) values. Quantitation of expression was calculated relative to the endogenous control (18S). All experiments were performed in triplicate with three independent experiments.

### Animal studies

All animal experiments were approved by the Peter MacCallum Cancer Centre Animal Experimentation Ethics Committee (E556 and E628) and conducted in accordance with the National Health and Medical Research Council Australian Code of Practice for the Care and Use of Animals for Scientific Purposes. The mice were maintained in individually ventilated cages enriched with bedding, nesting material, carboard and wood tunnels. Housing room temperature ranges from 19 to 21 °C. The facility runs on a 10 h dark, 13 h light cycle with 2 × 30 min of dimmed light to mimic dawn and dusk. Their diet comprised irradiated mice cubes (Ridley). The mice are provided with reverse osmosis (RO) water with a chlorine residual of 2.0–4.0 ppm via an automatic watering system (Avidity Edstrom System).

C57BL/6 as well as immune-compromised C57BL/6 RAG1^-/-^ and C57BL/6 RAG2^-/-^γc^-/-^ strains were used for the AT3OVA model. The AT3 and 4T1ch9 model were assessed in C57BL/6 and BALB/c mice, respectively. For all models, tumour cells (AT3 and AT3OVA: 5 × 10^5^; 4T1ch9: 5 × 10^4^) were suspended in PBS and injected into the fourth mammary fat pads of 6- to 8-week-old female mice while under anaesthesia using isoflorane. Tumour volume (length × width^2^ × 0.5) was assessed by calliper measurements every 3–4 days. Once tumours reached an average of 20–40 mm^2^, mice were randomised into treatment groups of indicated sizes to commence drug treatment (day 1). Indicated doses of olaparib and AZD1775 were administered via oral gavage daily 5 out of 7 days a week throughout the duration of the experiment. 200 µg of anti-PD-1 was administered via intraperitoneal injections (AT3 and AT3OVA: days 1, 5, 8 and 12. 4T1ch9: days 1 and 5). 40 µg of ADU-S100 was administered via intra-tumoral injections on days 1, 3, 5 for all tumour models. The immune depletion antibodies were administered on day 0 (the day before targeted therapy treatment), day 1 (the first day of targeted-therapy treatment), day 7, 14 and 21.

Mice were euthanized using carbon dioxide (Euthanex Chamber) or cervical dislocation if the tumours reached ethical limit of 1400 mm^3^ or if the animals displayed health indicators that met the institutional criteria for sacrifice.

### Tumour collection and preparation for flow cytometry analysis of tumour-infiltrating immune subsets

The mice are euthanized via cervical dislocation. Tumours were excised and digested using a mix of 1 mg/ml collagenase type IV (Sigma-Aldrich) and 0.02 mg/ml DNAase (Sigma-Aldrich). After 30 min of digestion at 37 °C, cells were passed through a 70 µm filter twice. For analysis of intracellular IFNγ, cells were stimulated with 50 ng/ml phorbol 12-myristate 13-acetate (PMA; Sigma-Aldrich), 1 µg/ml Ionomycin (Sigma-Aldrich), GolgiSTOP (1:1500 dilution; Becton Dickinson) and GolgiPLUG (1:1000 dilution; Becton Dickinson) for 4 h at 37 °C. Single-cell suspensions were then stained with the appropriate antibodies and analysed by flow cytometry analysis on LSR II, BD LSRFortessa™ or FACSymphony™ flow cytometers (BD Biosciences). FACS data was analysed using FlowJo version 10 software (Tree Star Inc., USA). The antibodies used here: CD45.2 (clone: 104; 1:200 dilution, 109824, Biolegend), TCRβ (1:200 or 1:400 dilution; clone: H57-597; 563221, BD Biosciences; 109241, 109226, Biolegend; 12-5961-83, 17-5961-83, 11-5961-85, 45-5961-82, eBioscience), CD4 (clone: RM4-5; 1: 400 dilution; 100551, Biolegend; clone: Gk1.5; 1:200 dilution; 25-0041-82, eBioscience), CD8a (clone: 53-6.7; 1:400 dilution; 100748, Biolegend), CD11b (clone: M1/70; 1:400 dilution; 101242, Biolegend), CD11c (clone: N418; 1:400 dilution; 117335, Biolegend; 1:200 dilution; 53-0114-82, eBioscience), CD279 (PD-1; clone: J43; 1:200 dilution; 11-9985-85, eBioscience), FOXP3 (clone: FJK-16s; 1: 200 dilution; 48-5773-82, eBioscience), Granzyme B (clone: GB11; 1:200 dilution; 515406, BioLegend; clone: NGZB; 1:100 dilution; 25-8898-82, eBioscience), Ki67 (clone: 16A8; 1:200 dilution; 652408, Biolegend), IFNγ (clone: XMG1.2; 1:200 dilution; 12-7311-82, eBioscience; 505839, Biolegend), CD83 (clone: Michael-19; 1:200 dilution; 121515, 1215158, Biolegend), CD103 (clone: 2E7, 1:200 dilution; 46-1031-82, 1:400 dilution; 12-1031-83, eBioscience), CD274 (B7-H1/PD-L1, clone: MIH5, 1:200 dilution; 25-5982-82, 1:400 dilution: 12-5982-82, eBioscience), MHCII (1 A/1E, clone: M5/114.15.2; 1:400 dilution; 17-5321-82; eBioscience, 1:200 dilution; 107620, Biolegend), CD80 (clone: 16-10A1, 1:400 dilution; 104729, Biolegend), CD86 (clone Gl1, 1:400 dilution; 105028, 105037, Biolegend), MHCI (H2Kb; clone: AF6-88.5; 1:400 dilution; 116520, Biolegend), MHCI (H2Db, clone: 28-14-8, 1:200 dilution; 48-5999-82, eBioscience), MHCI (H2Kb/H2Db, clone: 28.8.6, 1:400 dilution; 114612, Biolegend). LIVE/DEAD™ Fixable Yellow Dead cell stain kit and LIVE/DEAD™ Fixable Blue Dead cell stain kit (ThermoFisher Scientific) were used as viability markers. The gating strategy is provided in Supplementary Figs. 12 and 13.

### Quantification and statistical analysis

Two-sided Student’s t-test or one-way analysis of variance (ANOVA) with Tukey’s multiple comparison test was used to compare between treatment groups. Tumour growth curves were analysed using two-way ANOVA with Tukey’s multiple comparison test. Survival differences between treatment groups in vivo were determined using log-rank (Mantel-Cox) analysis. Statistical analyses (not including differential gene expression or GSEA analyses) were performed using Prism 7-9 (GraphPad). A two-tailed *p* < 0.05 was considered statistically significant.

### Reporting summary

Further information on research design is available in the Nature Research Reporting Summary linked to this article.

### Supplementary information

Supplementary Figures and Tables

Reporting Summary

## Data Availability

Transcriptomic data from this study have been deposited in the Sequence Read Archive and can be accessed via this link: Accession code: PRJNA864115. Additional relevant data that support the findings of this study are available from the corresponding author upon reasonable request.
