# Supplementary material for: Combined PARP and WEE1 inhibition triggers anti-tumor immune response in BRCA1/2 wildtype triple-negative breast cancer
Source: NPJ Breast Cancer. 2023 Aug 15;9:68. doi: 10.1038/s41523-023-00568-5 (PMC10427618; doi:10.1038/s41523-023-00568-5)
Supplement: Supplementary file 1 — Supplementary Figures and Tables [file 41523_2023_568_MOESM1_ESM.pdf]

a

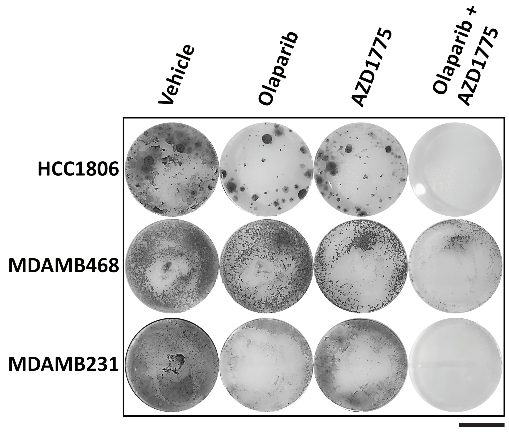

b

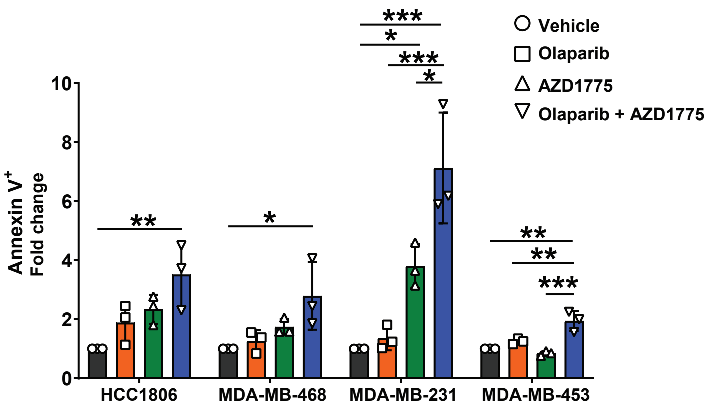

c

HCC1806 72 hr Olaparib vs Vehicle				
GENESET	Size	NES	NP	FDR
HALLMARK_INTERFERON_ALPHA_RESPONSE	83	1.598	0.002	0.105
HALLMARK_IL2_STAT5_SIGNALING	133	1.541	0	0.099
HALLMARK_INTERFERON_GAMMA_RESPONSE	145	1.443	0.015	0.171

HCC1806 72 hr AZD1775 vs Vehicle				
GENESET	Size	NES	NP	FDR
HALLMARK_INTERFERON_ALPHA_RESPONSE	83	1.619	0	0.079
HALLMARK_INTERFERON_GAMMA_RESPONSE	145	1.544	0.005	0.08
HALLMARK_G2M_CHECKPOINT	195	1.485	0	0.084
HALLMARK_UNFOLDED_PROTEIN_RESPONSE	109	1.481	0.002	0.065

MDA-MB-231 72 hr Olaparib vs Vehicle				
GENESET	Size	NES	NP	FDR
HALLMARK_INTERFERON_ALPHA_RESPONSE	83	1.787	0	0.017
HALLMARK_INTERFERON_GAMMA_RESPONSE	145	1.472	0.006	0.199

MDA-MB-231 72 hr AZD1775 vs Vehicle				
GENESET	Size	NES	NP	FDR
HALLMARK_KRAS_SIGNALING_UP	103	1.519	0.009	0.225
HALLMARK_INFLAMMATORY_RESPONSE	99	1.499	0.009	0.136
HALLMARK_TNFA_SIGNALING_VIA_NFKB	161	1.46	0	0.123

d

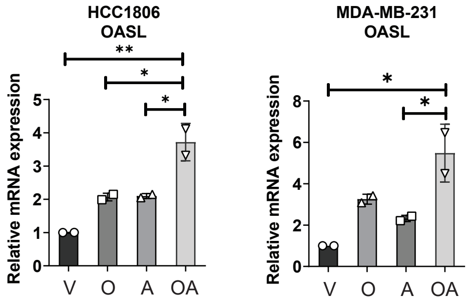

e

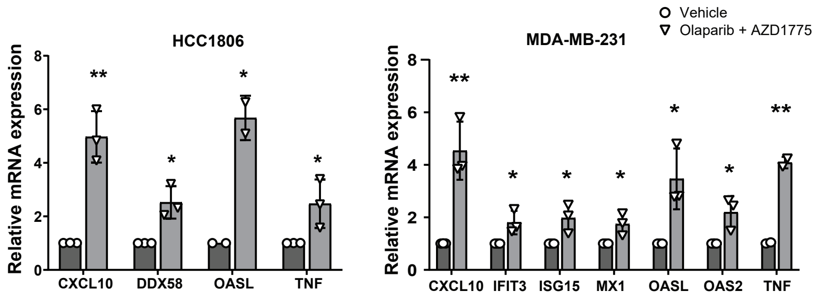

f

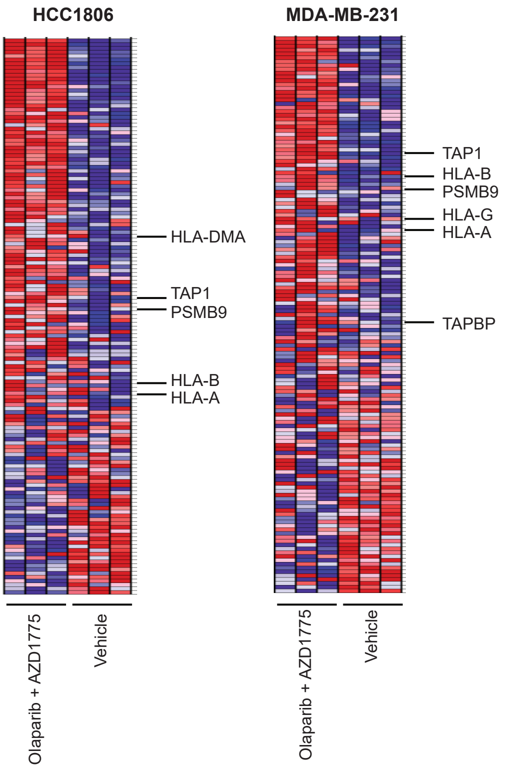

g

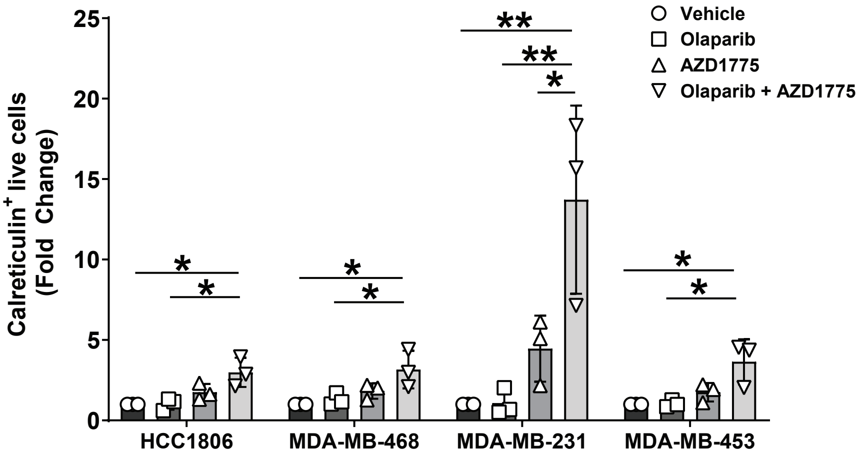

Supplementary Figure 1

a. Clonogenic assays of TNBC cell lines grown continuously for 2 weeks in vehicle (DMSO), olaparib and/or AZD1775. Scale bar represents 1.75 cm. **b.** Apoptosis assay via assessment of Annexin V and propidium iodide expression in TNBC cell lines. Data shows the fold change ± s.d. of apoptotic (Annexin V positive) cells in each group relative to the vehicle treated group. **c.** Top ranked, upregulated GSEA Hallmarks in HCC1806 and MDA-MB-231 cells treated in vitro for 72 hours with olaparib or AZD1775. Normalized p<0.05, FDR<0.25. **d** and **e.** Gene expression via qRT-PCR in HCC1806 and MDA-MB-231 cells after 72 hours treatment with olaparib and/or AZD1775. Data shows mean relative mRNA expression ± s.d.. **f.** Heatmaps for the Hallmark gene set of interferon gamma response by GSEA. Major histocompatibility complex genes and genes involved in antigen presentation are as indicated. **g.** TNBC cells were treated with olaparib and AZD1775 and analysed for calreticulin expression via flow cytometry. Data depicts mean fold change of percentage calreticulin positive, live cells relative to vehicle controls ± s.d.. * P <0.05; ** P <0.01; *** P <0.001; **** P <0.0001 by one-way ANOVA or Student t-test.

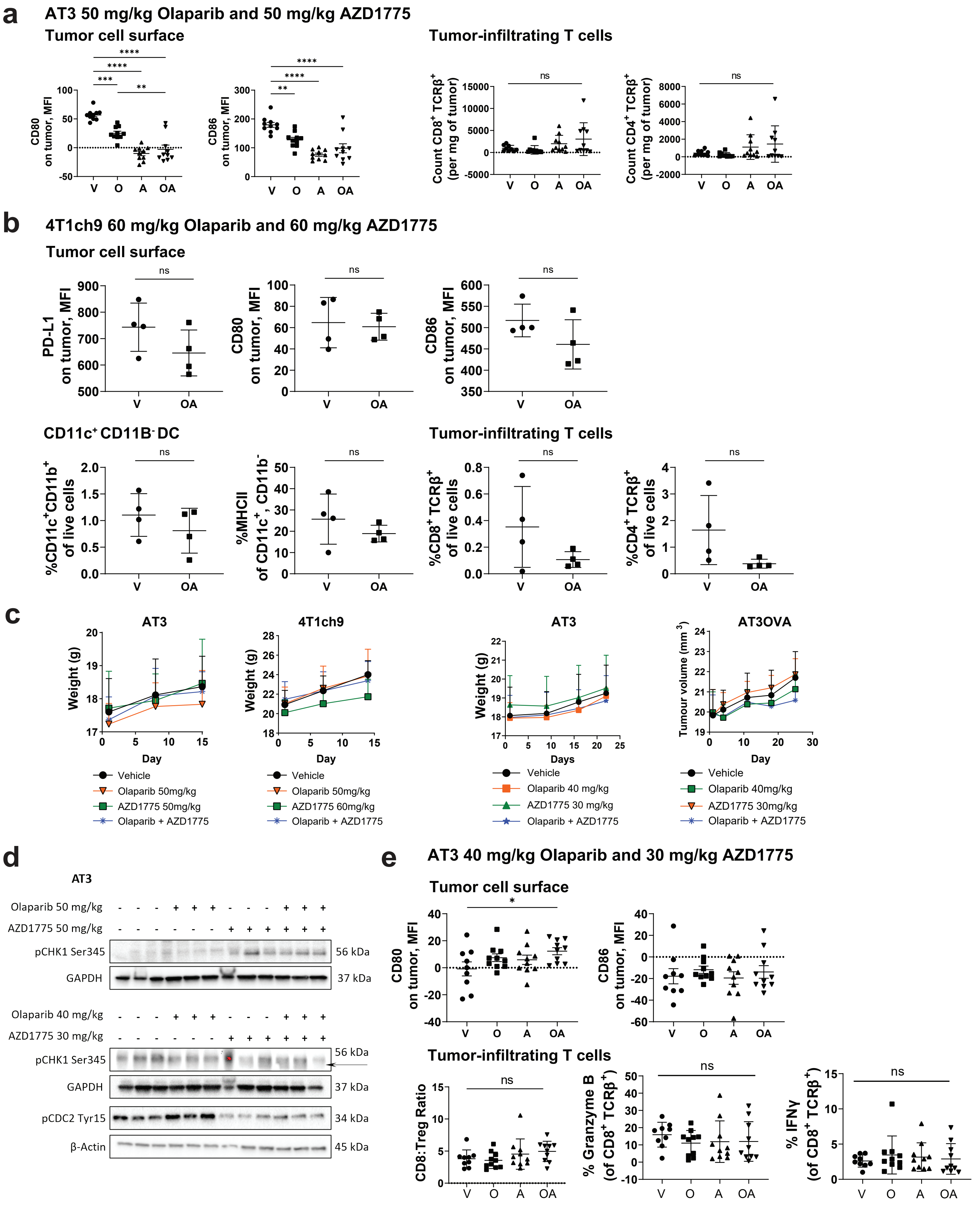

Supplementary Figure 2

a. AT3 tumor-bearing mice were treated with vehicle, olaparib and/or AZD1775 for 15 days. Tumors and were harvested and analysed via flow cytometry. 10 mice per treatment group. Data shows mean ± s.d.. **b.** 4T1ch9 tumor-bearing mice were treated with vehicle, olaparib and/or AZD1775 for 15 days. Tumors were harvested and analysed via flow cytometry. 4 mice per treatment group. Data shows mean ± s.d.. **c.** Weights of AT3, AT3OVA, and 4T1ch9 tumor-bearing mice treated with the indicated compounds. Data shows mean weight ± s.d.. **d.** Assessing protein expression of DNA damage and cell cycle markers via Western blot after 72 hours of indicated treatment on AT3 tumor-bearing mice. **e.** AT3 tumor-bearing mice were treated with vehicle, olaparib and/or AZD1775 for 15 days. Tumors and were harvested and analysed via flow cytometry. 10 mice per treatment group. Data shows mean ± s.d.. * P <0.05; ** P <0.01; *** P <0.001; **** P <0.0001 by one-way ANOVA.

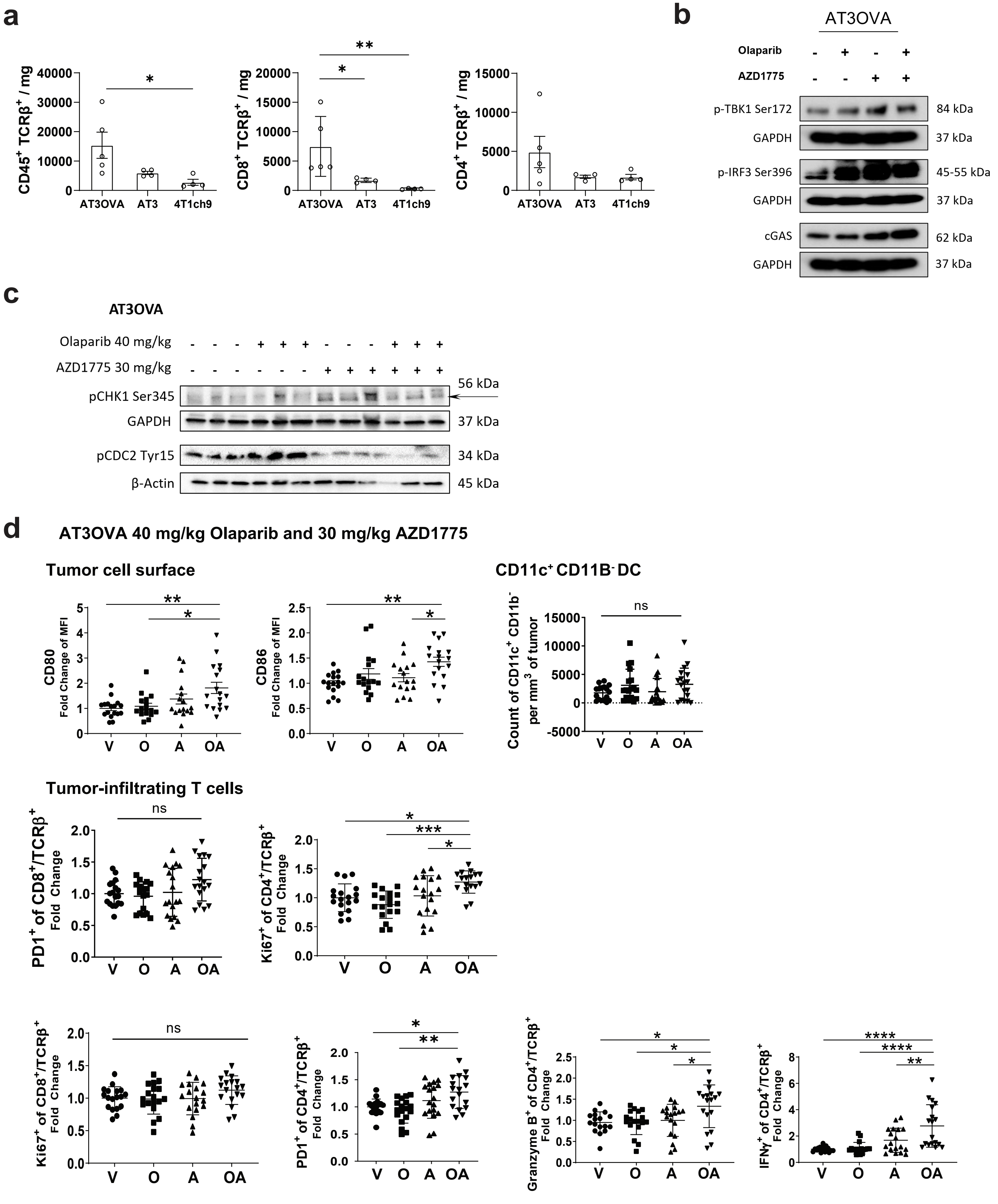

Supplementary Figure 3

a. AT3OVA (n=6), AT3 (n=4) and 4T1ch9 (n=5) tumor-bearing mice were sacrificed on day 8 (4T1ch9) or day 11 (AT3OVA and AT3) post injection of tumor cells. Tumors were analysed via flow cytometry for the indicated immune cell populations. **b.** Assessing protein expression of components of the STING pathway via Western blot after 24 hours of treatment. Results are representative of at least 2 independent experiments. **c.** Assessing protein expression of DNA damage and cell cycle markers via Western blot after 72 hours of indicated treatment on AT3OVA tumor-bearing mice. **d.** AT3OVA tumor-bearing mice were treated with vehicle, olaparib and/or AZD1775 for 16 days. Tumors and were harvested and analysed via flow cytometry. 6 mice per treatment group. Data pooled from 3 independent experiments. Data shows mean ± s.d.. * P <0.05; ** P <0.01; *** P <0.001; **** P <0.0001 by one-way ANOVA.

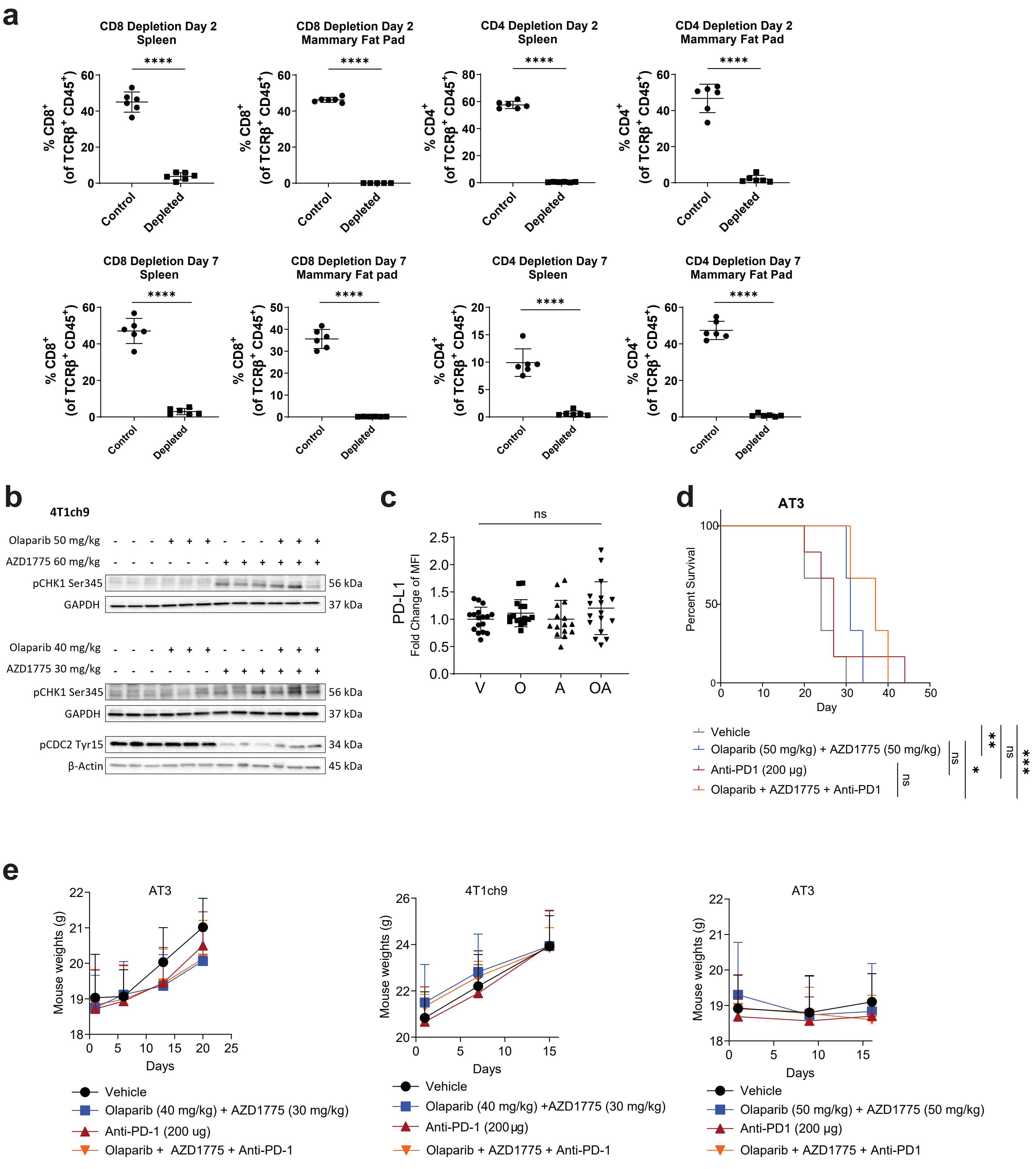

Supplementary Figure 4

a. Percentage of CD8 and CD4 T cells in the spleen and mammary fat pad of naïve C57BL/6 mice 2 and 7 days after CD8 and CD4 T cell anti-body depletion. Data shows mean ± s.d.. **b.** Assessing protein expression of DNA damage and cell cycle markers via Western blot after 72 hours of indicated treatment on 4T1ch9 tumor-bearing mice. **c.** AT3OVA tumor-bearing mice were treated with vehicle, olaparib and/or AZD1775 for 16 days. Tumors and were harvested and analysed via flow cytometry. 6 mice per treatment group. Data pooled from 3 independent experiments. Data shows mean ± s.d.. **d.** Survival curves of AT3 tumor bearing mice treated with olaparib, AZD1775, and/or anti-PD-1 (clone: RMP1-14). 6 mice per treatment group. **e.** Weights of AT3 and 4T1ch9 tumor-bearing mice treated with the indicated compounds. Data shows mean weight ± s.d.. *, *P* <0.05; **, *P* <0.01; ***, *P* <0.001; ****, *P* <0.0001 by Student t-test and log-rank (Mantel-Cox) test for survival curves.

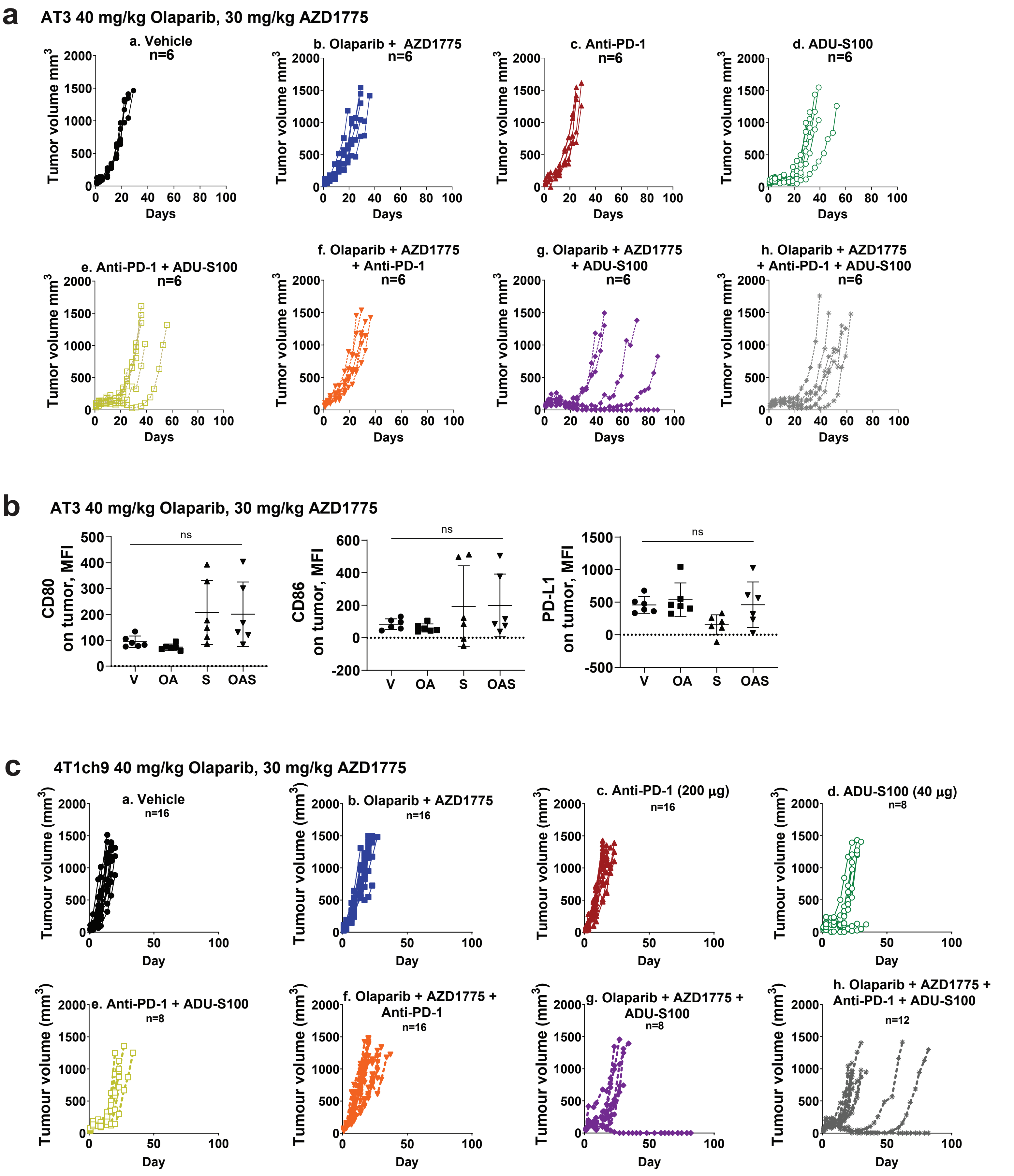

Supplementary Figure 5

a. Tumor growth curves of individual mice bearing AT3OVA tumors treated with indicated compounds. **b**. AT3 tumor-bearing mice were treated with vehicle, olaparib, AZD1775, and/or ADU-S100 for 15 days. Tumors were harvested and analysed via flow cytometry. 6 mice per treatment group. Data shows mean ± s.d.. **c.** Tumor growth curves of individual mice bearing 4T1ch9 tumors treated with indicated compounds. 8 mice per treatment group. Data pooled from 2 independent experiments.

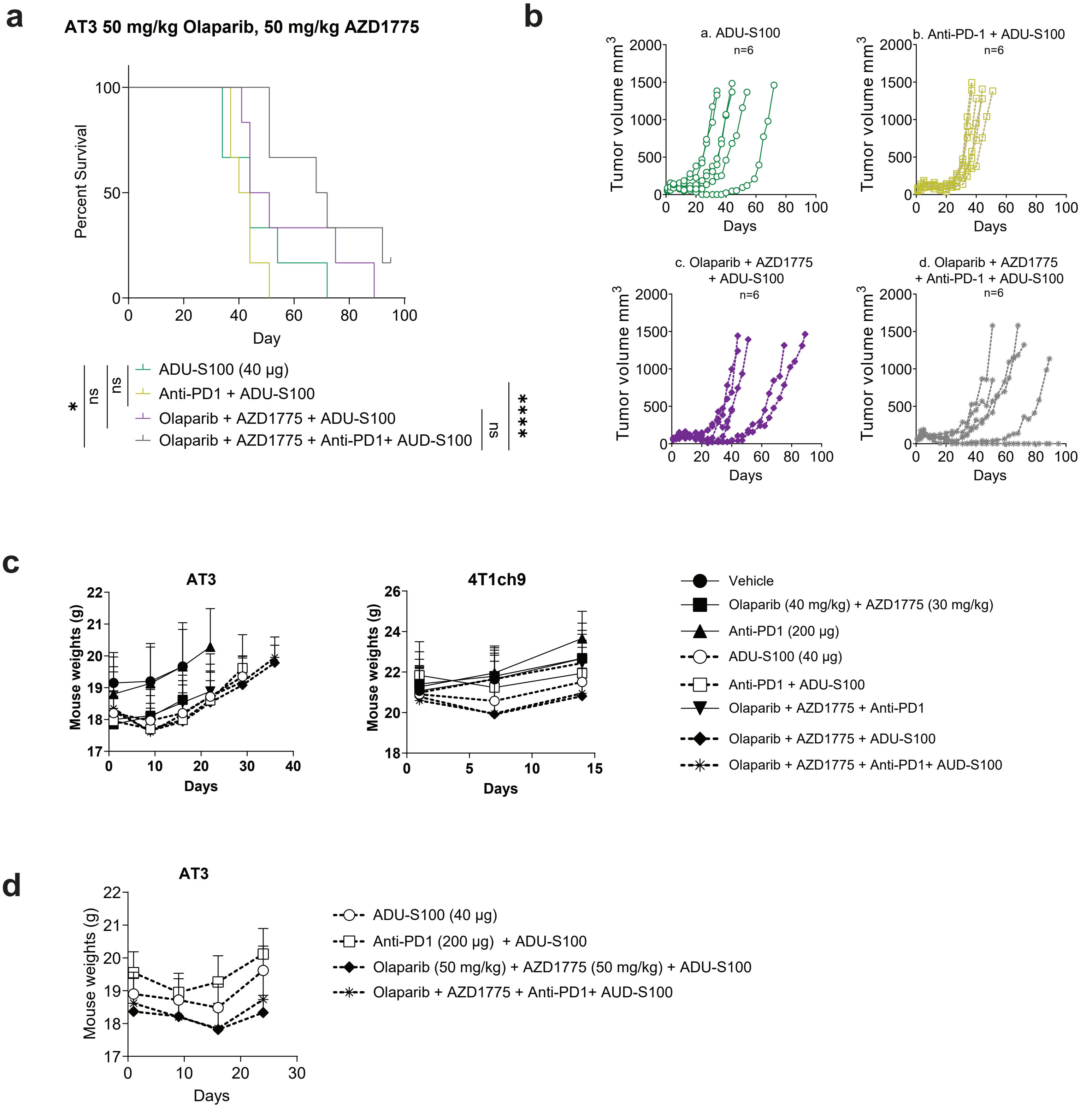

Supplementary Figure 6

a. Survival curves of AT3 tumor bearing mice treated with olaparib, AZD1775, ADU-S100 and/or anti-PD-1 (clone: RMP1-14). 6 mice per treatment group. **b.** Tumor growth curves of individual mice bearing AT3 tumors treated with indicated compounds. 6 mice per treatment group. **c.** Weights of AT3, and 4T1ch9 tumor-bearing mice treated with vehicle, Olaparib (40 mg/kg), AZD1775(30 mg/kg), anti-PD-1 (200 µg) and/or ADU-S100 (40 µg). Data shows mean weight ± s.d.. **d.** Weights of AT3 tumor-bearing mice treated with vehicle, Olaparib (50 mg/kg), AZD1775(50 mg/kg), anti-PD-1 (200 µg) and/or ADU-S100 (40 µg).. Data shows mean weight ± s.d.. *, P <0.05; **, P <0.01; ***, P <0.001; ****, P <0.0001 by log-rank (Mantel-Cox) test for survival curves.

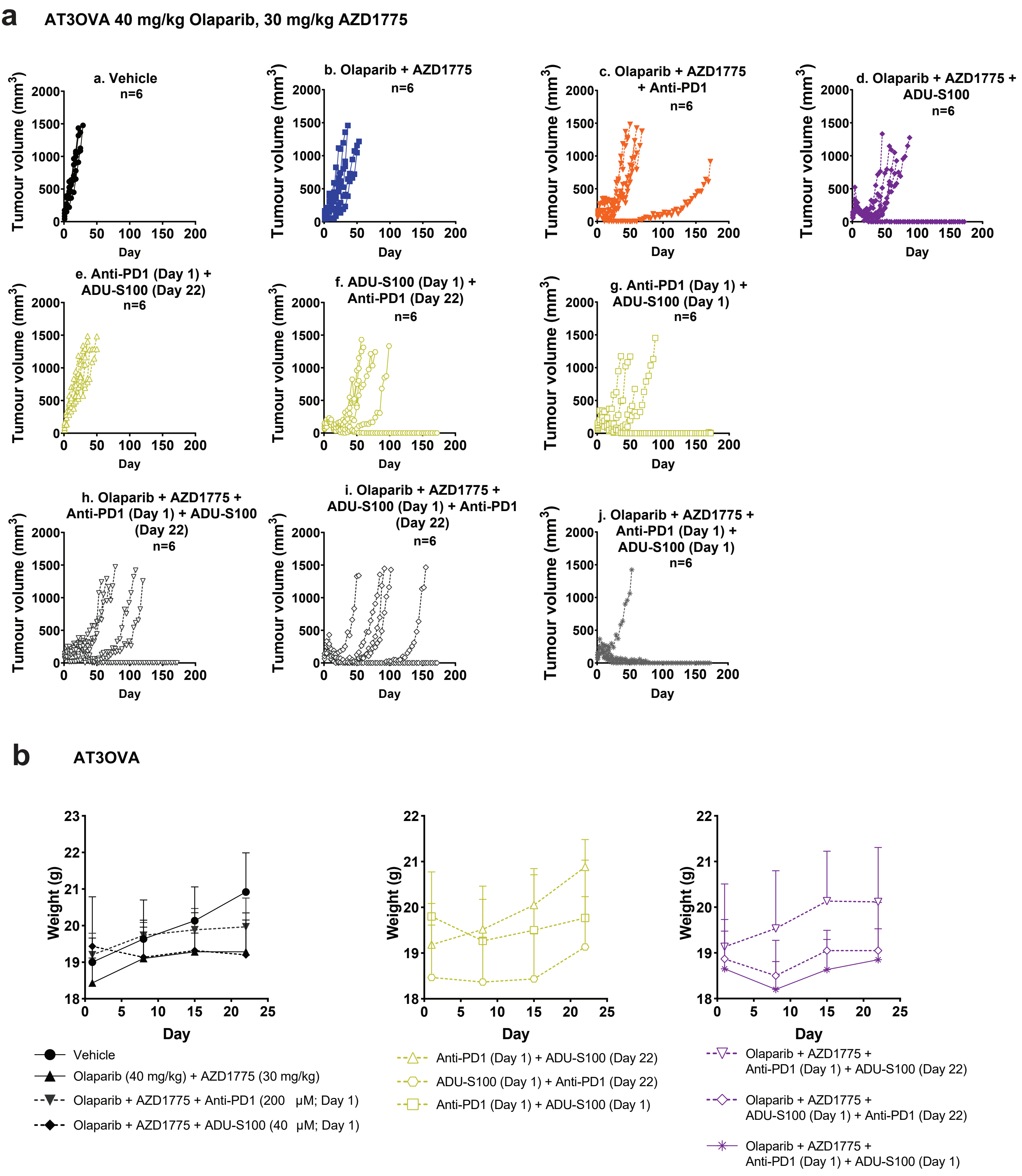

Supplementary Figure 7

a. Tumor growth curves of individual mice bearing AT3OVA tumors treated with indicated compounds. 6 mice per treatment group.

b. Weights of AT3OVA tumor-bearing mice treated with the indicated compounds. Data shows mean weight ± s.d..

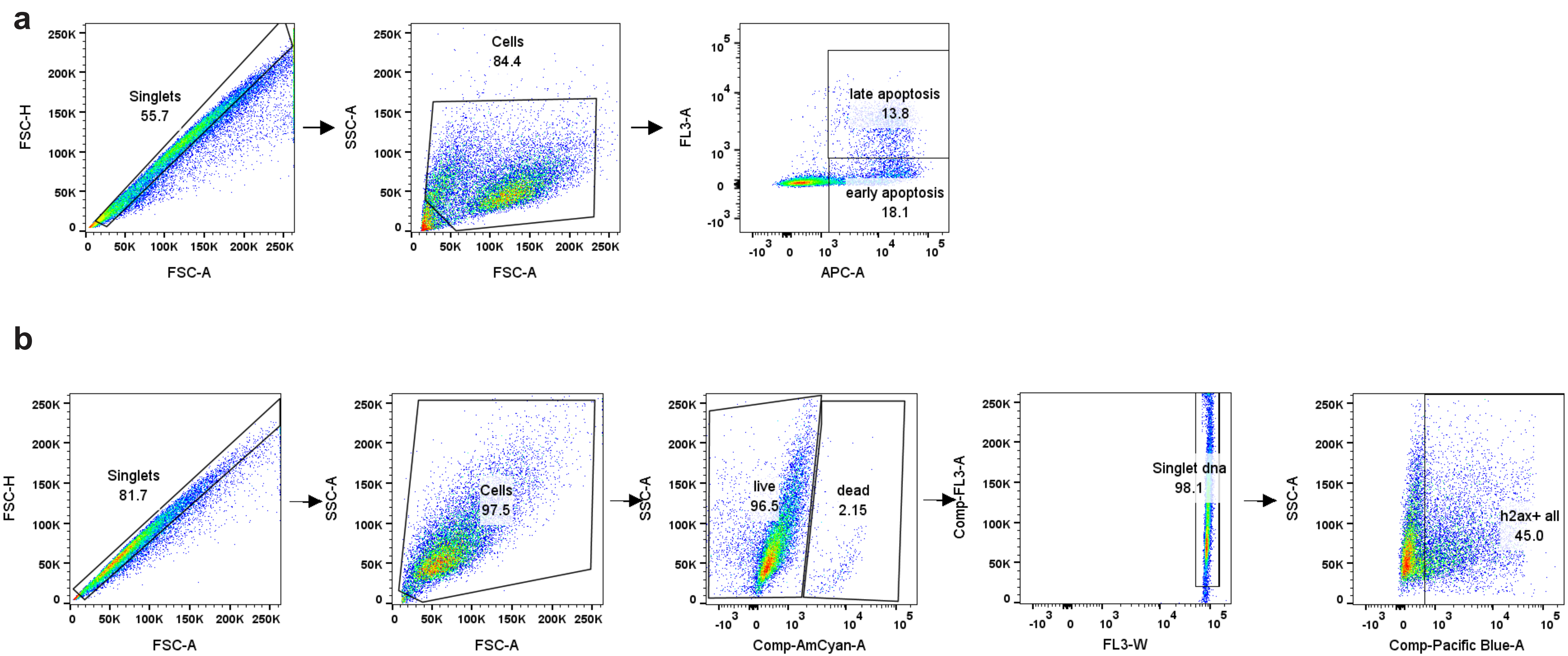

Supplementary Figure 8

Gating strategies used in flow cytometric analysis of tumor cell lines. Debris and non-viable cells were gated out. Live cells were analysed for the expression of **a.** AnnexinV positive cells, and **b.** **γ**H2AX positive. The singlet DNA gate in **b.** was used to gate out **γ**H2AX positive apoptotic cells.

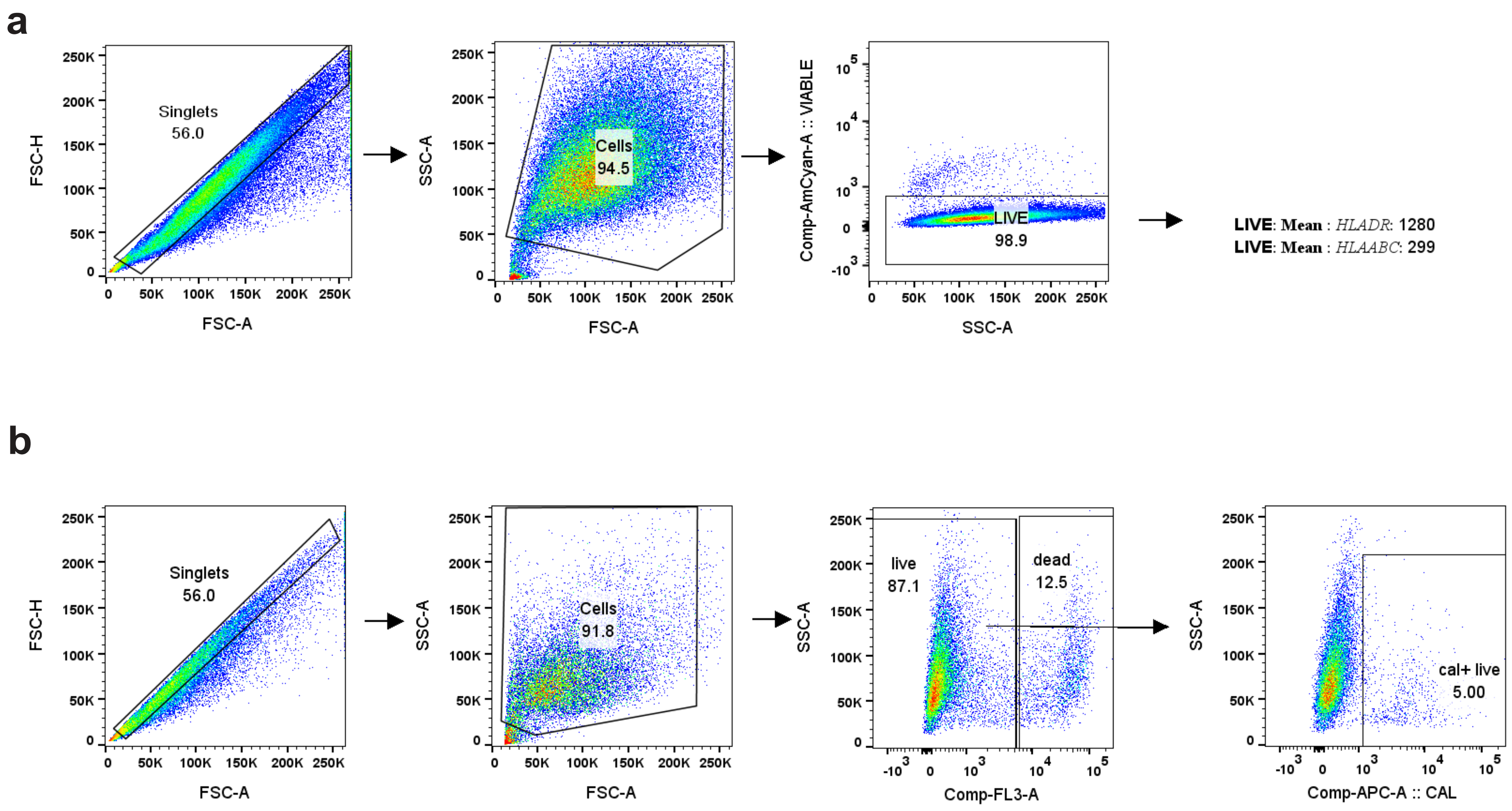

Supplementary Figure 9

Gating strategies used in flow cytometric analysis of tumor cell lines. Debris and non-viable cells were gated out. Live cells were analysed for the expression of **a.** major histocompatibility complexes I and II, and **b.** calreticulin positive cells.

Unprocessed immunoblots of Figure 1c

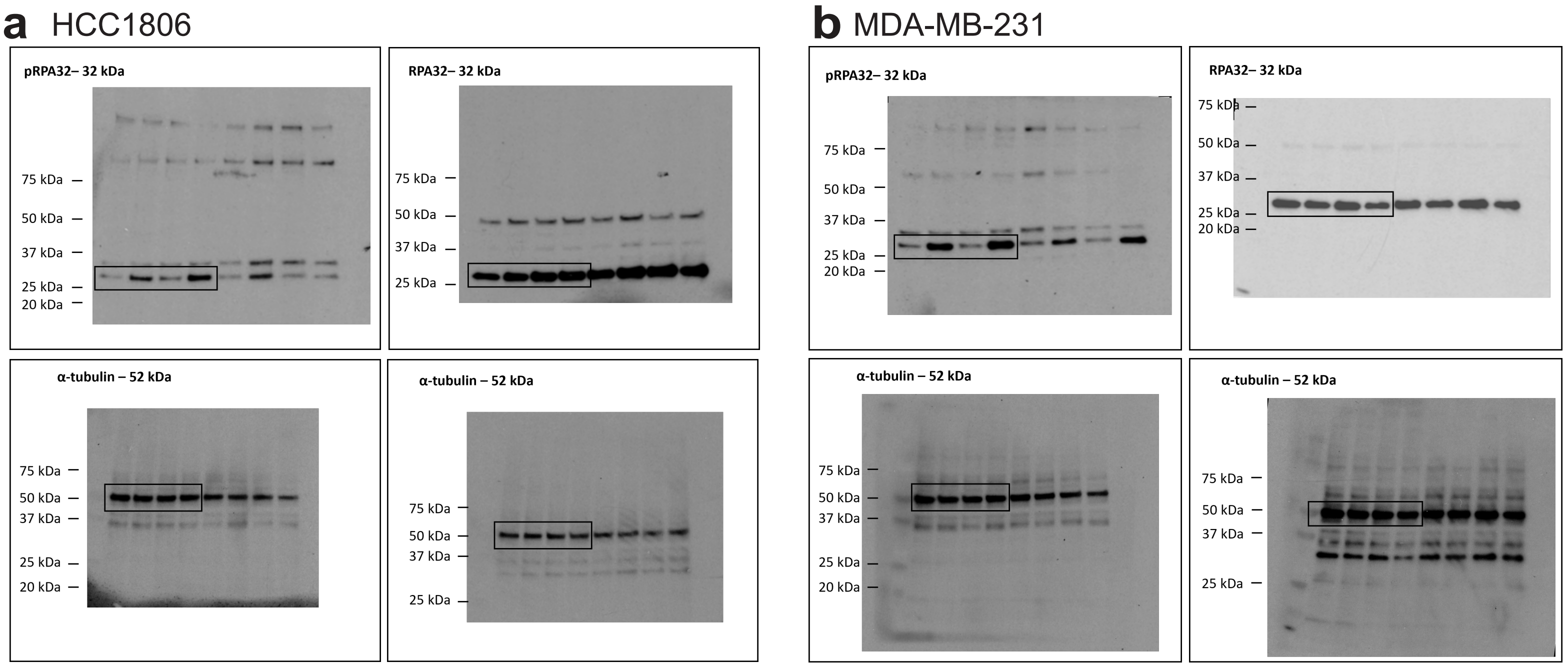

Unprocessed immunoblots of for Figure 1d

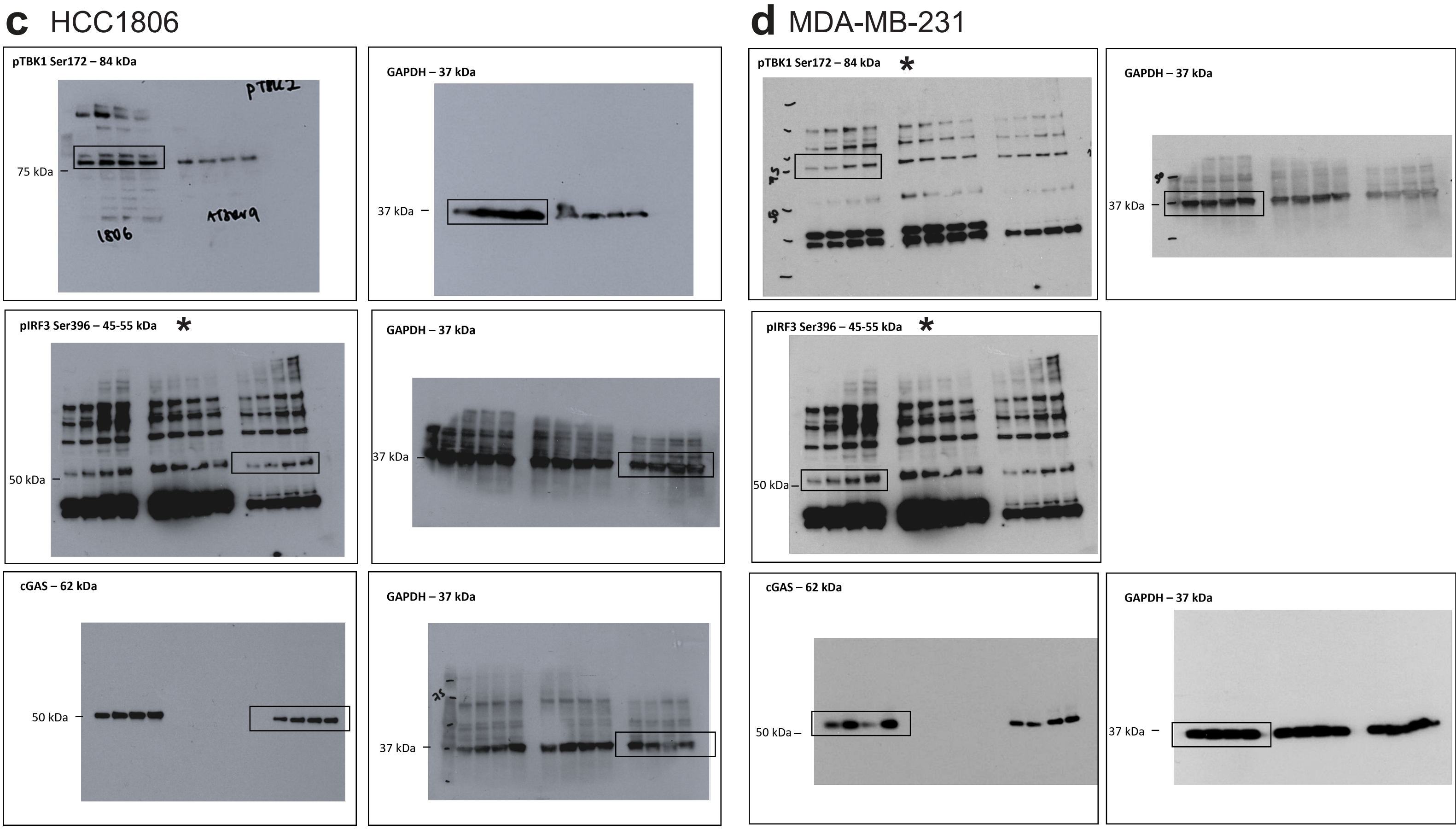

Supplementary Figure 10

Unprocessed immunoblots of **a - b.** Figure 1c, and **c - d.** Figure 1d. Immunoblot images marked * are images of the same blot, exposed to X-ray films for different times. This immunoblot was cut horizontally at the 75 kDa mark. The top half of the membrane was probed for pTBK1 Ser172 and the bottom half was probed for pIRF3 Ser396 and GAPDH (loading control).

a Unprocessed immunoblots of Supplementary Figure 2d

AT3 50 mg/kg Olaparib and 50 mg/kg AZD1775

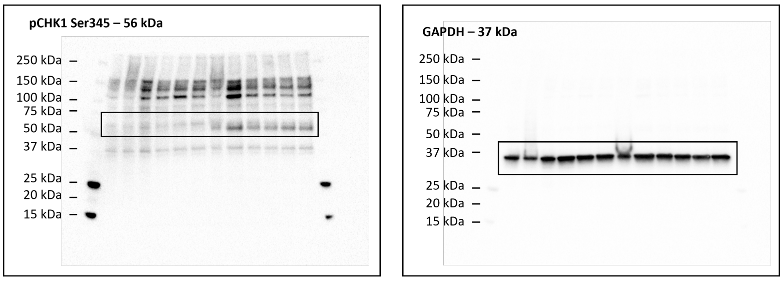

AT3 40 mg/kg Olaparib and 30 mg/kg AZD1775

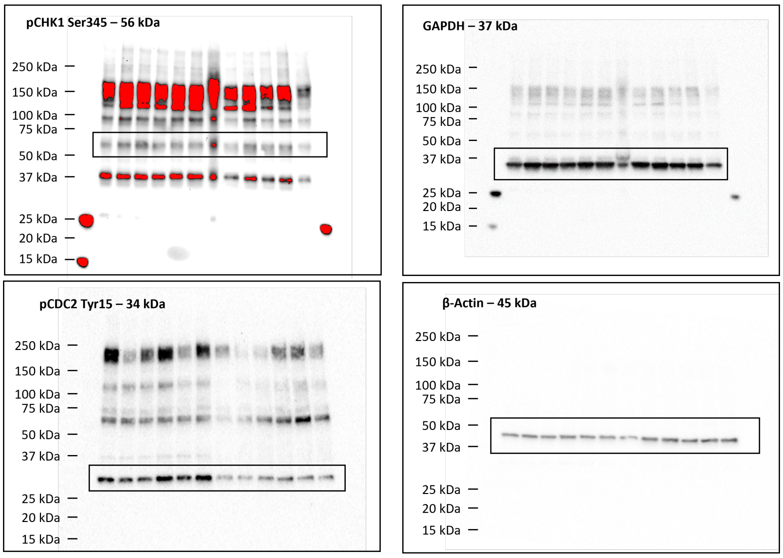

b Unprocessed immunoblots of Supplementary Figure 3b

AT3OVA *in vitro*

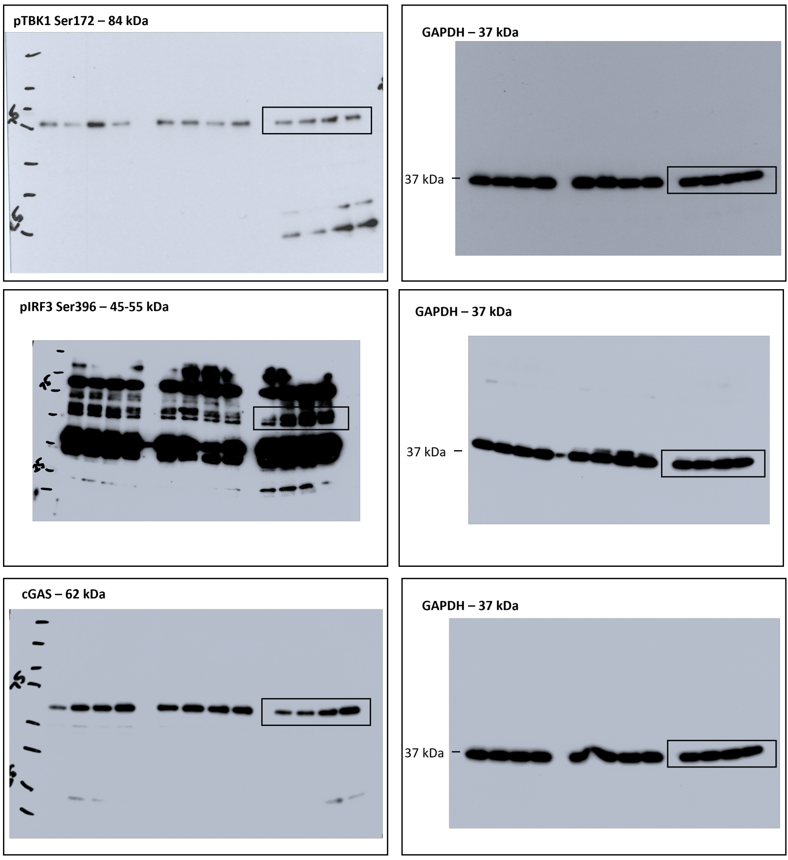

c Unprocessed immunoblots of Supplementary Figure 3c

AT3OVA 40 mg/kg Olaparib and 30 mg/kg AZD1775

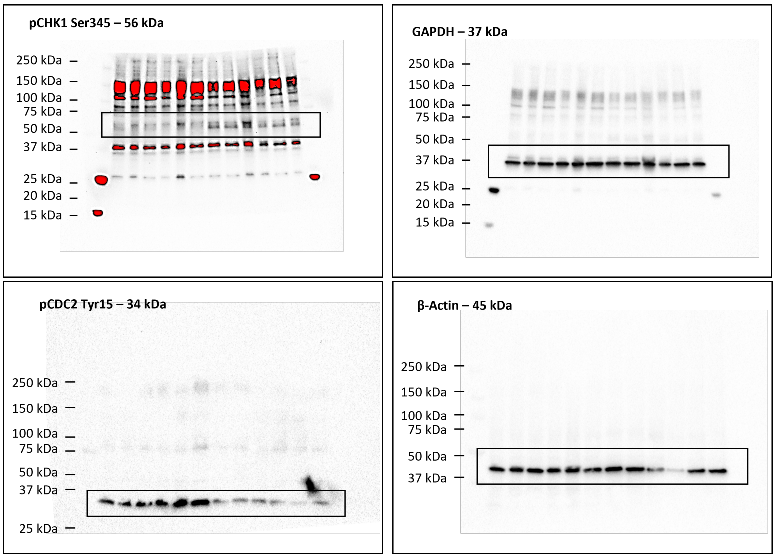

d Unprocessed immunoblots of Supplementary Figure 4b

4T1ch9 50 mg/kg Olaparib and 60 mg/kg AZD1775

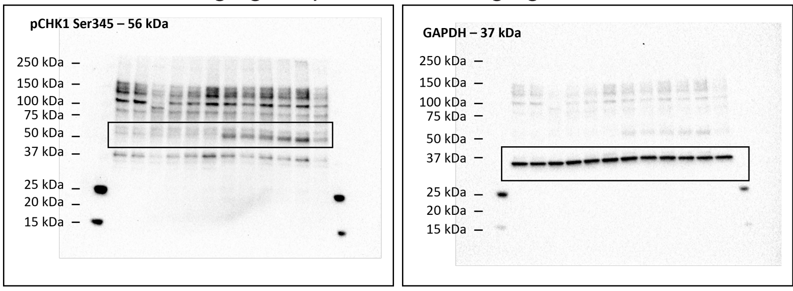

4T1ch9 40 mg/kg Olaparib and 30 mg/kg AZD1775

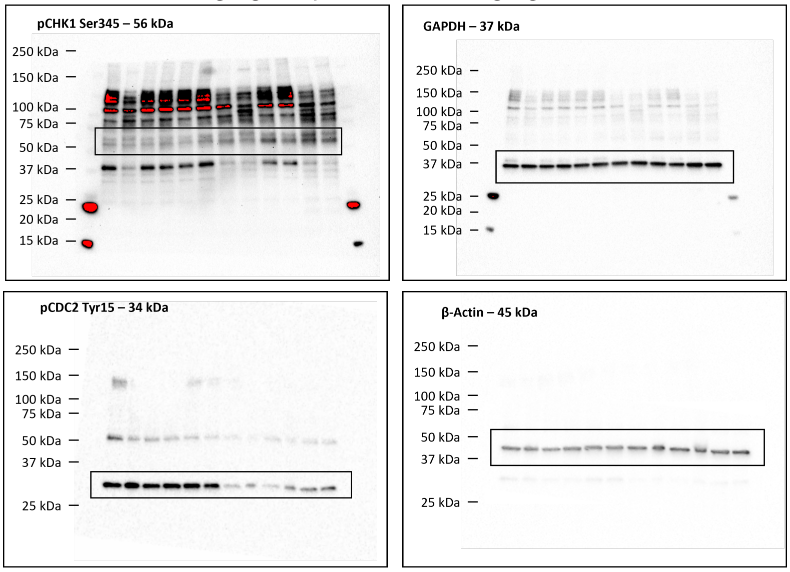

Supplementary Figure 11
Unprocessed immunoblots of **a.** Supplementary Figure 2d, **b.** Supplementary Figure 3b, **c.** Supplementary Figure 3c, and **d.** Supplementary Figure 4b.

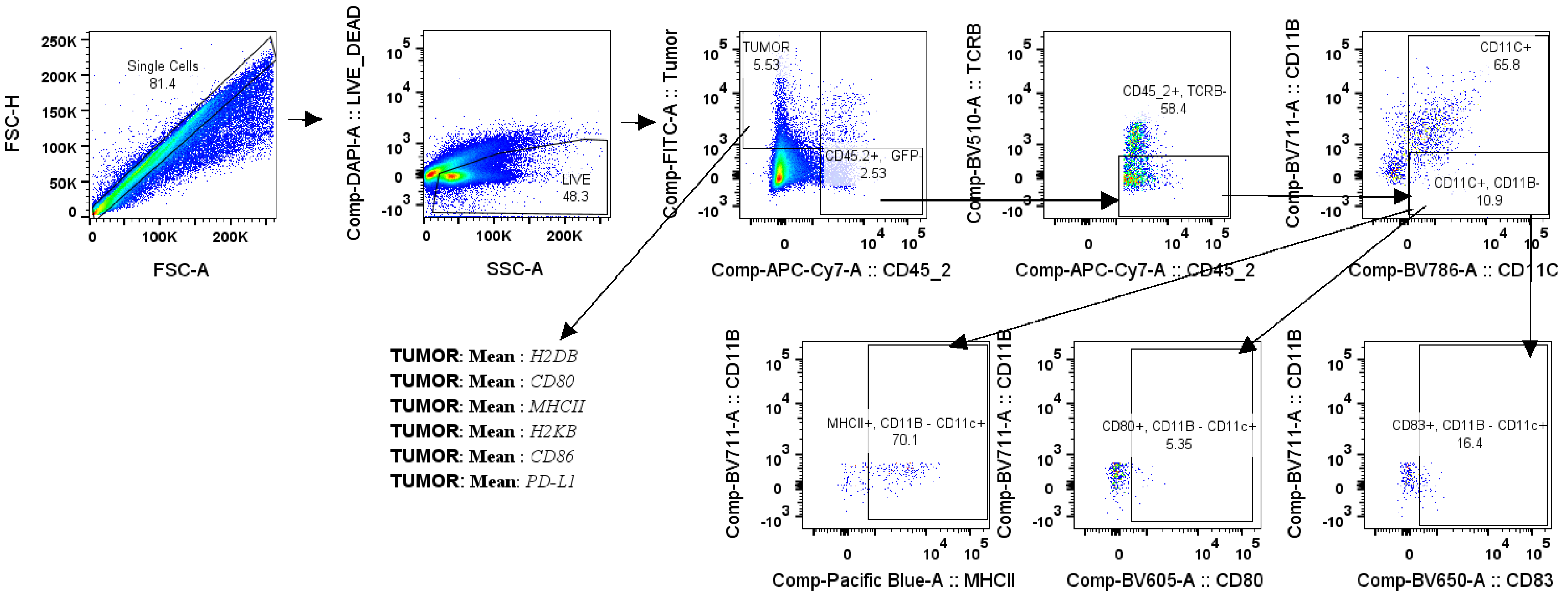

Supplementary Figure 12

Gating strategy used in flow cytometric analysis of harvested murine tumor cells. Debris and non-viable cells were gated out. Live cells were analysed for the expression of GFP+ tumor cells and GFP- non-tumor cells. The mean fluorescent intensities of H2Kb, H2Db (MHCI alleles), MHCII, CD80, CD86 and PD-L1 expression was analysed from the GFP+ tumor cells. CD45+, TCRβ- cells were identified off the GFP- cells. A CD11c vs CD11b plot was used to identify CD11c+, CD11b- dendritic cells out of CD45+, TCRβ- cells. Dendritic cell activation markers MHCII, CD80 and CD83 expression were analysed out of the CD11c+, CD11b- cells.

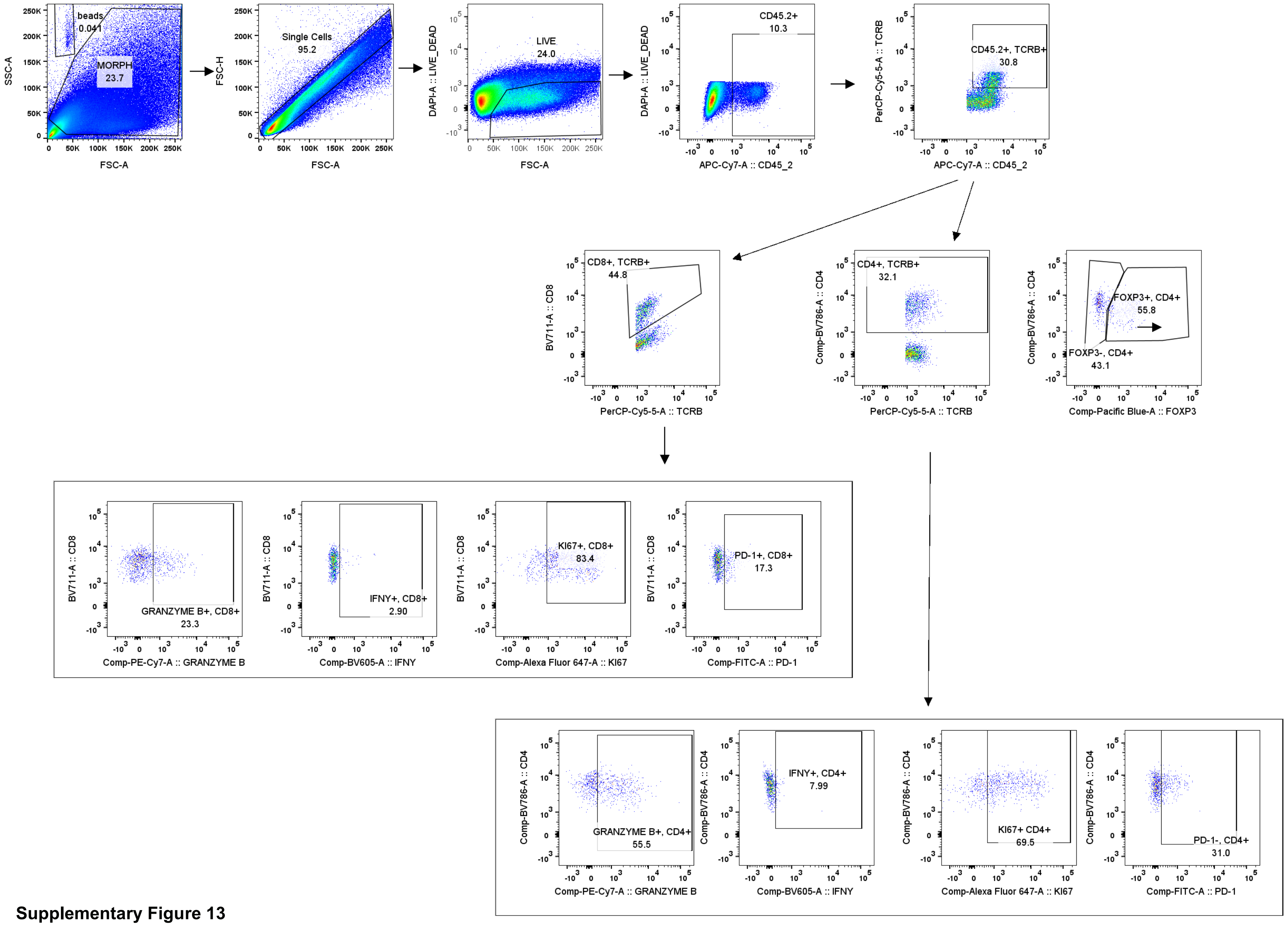

Supplementary Figure 13

Gating strategy used in flow cytometric analysis of T cells from harvested murine tumors, spleen or mammary fat pad. Debris and non-viable cells were gated out. Live cells were analysed for the expression of CD45+ cells and TCRβ+ cells were identified off that population. CD8 vs TCRβ and CD4 vs TCRβ plots were used to identify CD8+ and CD4+ T cells, respectively. FOXP3 (T regulatory cells) expression was analysed out of CD4 T cells. Granzyme B, IFNγ, Ki67 and PD-1 positive cells were analysed from CD8+ and CD4+ tumor-infiltrating T cells

Supplementary Table 1. Cell lines: Combination of Olaparib + AZD1775 vs Vehicle		
	**HCC1806**	**MDA-MB-231**
**Replication stress**		
pRPA32 Ser4/8	Increase	Increase
**STING pathway activation**		
pTBK1 Ser172	Increase	Increase
pIRF3 Ser396	Increase	Increase
cGAS	Increase	Increase
CXCL10 mRNA	Increase	Increase
IFNB1 mRNA	Increase	N/A
**Tumor cell surface**		
MHCI	NS	Increase
MHCII	NS	Increase
**Tumor cell surface + IFNγ Stimulation**		
MHCI	Increase	Increase
MHCII	Increase	Increase
N/A: Not applicable. NS: Non-significant		

Supplementary Table 2. AT3, AT3OVA, 4T1ch9: Combination of Olaparib + AZD1775 vs Vehicle

	AT3	AT3OVA	4T1ch9	AT3	AT3OVA	4T1ch9
	Olaparib 50 mg/kg - 60 mg/kg, AZD1775 50-60 mg/kg			Olaparib 40 mg/kg, AZD1775 30 mg/kg		
**Improve survival**	Yes	Not done	Yes	Yes	Yes	No
***In vivo* tumor cell surface markers**						
MHCI	Increase	Not done	Not done	Increase	Increase	Not done
PD-L1	Increase	Not done	NS	Decrease	Day 8 Increase; Day 16 NS	Not done
**Tumor-infiltrating immune cells**						
Number of CD8 T cells	NS	Not done	%; NS	Increase	NS	Not done
Number of CD4 T cells	NS	Not done	%; NS	NS	Increase	Not done
CD8:Treg Ratio	Increase	Not done	Not done	NS	Increase	Not done
% Granzyme B of CD8 T cells	NS	Not done	Not done	NS	Increase	Not done
% IFNγ of CD8 T cells	NS	Not done	Not done	NS	Increase	Not done
% Mature dendritic cells	Increase	Not done	NS	Increase	Increase	Not done
**Survival benefit with addition of anti-PD-1**	Yes	Not done	Not done	NS	Yes	NS
**Most efficacious drug combination***	O+A+S	Not done	Not done	O+A+S	O+A+S+P	O+A+S

*with the least number of drugs in the combination. O: Olaparib, A: AZD1775; S: STING agonist; P: Anti-PD-1

NS: Non-significant
