# Supplementary material for: Combined PARP and WEE1 inhibition triggers anti-tumor immune response in BRCA1/2 wildtype triple-negative breast cancer
Source: NPJ Breast Cancer. 2023 Aug 15;9:68. doi: 10.1038/s41523-023-00568-5 (PMC10427618; doi:10.1038/s41523-023-00568-5)
Supplement: Supplementary file 2 — Reporting Summary [file 41523_2023_568_MOESM2_ESM.pdf]

nature research

Corresponding author(s): Prof Sherene Loi

Last updated by author(s): 29/06/2023

Reporting Summary

Nature Research wishes to improve the reproducibility of the work that we publish. This form provides structure for consistency and transparency in reporting. For further information on Nature Research policies, see our Editorial Policies and the Editorial Policy Checklist.

Statistics

For all statistical analyses, confirm that the following items are present in the figure legend, table legend, main text, or Methods section.

n/a	Confirmed	
☐	☒	The exact sample size (*n*) for each experimental group/condition, given as a discrete number and unit of measurement
☐	☒	A statement on whether measurements were taken from distinct samples or whether the same sample was measured repeatedly
☐	☒	The statistical test(s) used AND whether they are one- or two-sided<br>*Only common tests should be described solely by name; describe more complex techniques in the Methods section.*
☒	☐	A description of all covariates tested
☐	☒	A description of any assumptions or corrections, such as tests of normality and adjustment for multiple comparisons
☐	☒	A full description of the statistical parameters including central tendency (e.g. means) or other basic estimates (e.g. regression coefficient) AND variation (e.g. standard deviation) or associated estimates of uncertainty (e.g. confidence intervals)
☐	☒	For null hypothesis testing, the test statistic (e.g. *F*, *t*, *r*) with confidence intervals, effect sizes, degrees of freedom and *P* value noted<br>*Give P values as exact values whenever suitable.*
☒	☐	For Bayesian analysis, information on the choice of priors and Markov chain Monte Carlo settings
☒	☐	For hierarchical and complex designs, identification of the appropriate level for tests and full reporting of outcomes
☒	☐	Estimates of effect sizes (e.g. Cohen's *d*, Pearson's *r*), indicating how they were calculated

Our web collection on statistics for biologists contains articles on many of the points above.

Software and code

Policy information about availability of computer code

Data collection

1. Quantitation of ATP present via CellTitre-Glo® Assay (Promega) luminescence read using Cytation™ 5 (BioTek).
2. Flow cytometric data were collected using BD Biosciences LSRII and BD FACSymphony cell analysers.
3. Immnofluorescence images were acquired using an Olympus Fluoview FV3000 confocal microscope system.
4. 3' mRNA-Sequencing was performed on NextSeq500 (Illumina).
5. qRT-PCR was performed on the StepOne Plus Real Time PCR machine (Applied Biosystem).
6. Immunoblot images were captured via X-ray film or ChemiDoc™ MP Imaging System (BioRad).

Data analysis

1. Curve fitting for CellTitre-Glo® Assay (Promega) was performed using GraphPad Prism software (versions 7 to 9).
2. Synergy quantitation for drug combination studies performed according to the Chou-Talalay method was analysed using CalcuSyn 2.0 (Biosoft).
3. FACS data was analysed using FlowJo version 10 software (Tree Star Inc., USA).
4. Western blot images were analysed using Image Lab software (version 6.0.1 build 34)
5. Gene Set Enrichment Analysis (GSEA) was performed using the GSEA software (version 2-2.2.4)
6. StepOne Plus software analysis Version 2 was used to calculate comparative Ct (ΔΔCt) (relative quantitation) values for qRT-PCR.
7. Statistical analyses were performed with GraphPad Prism software (versions 7 to 9). Two-sided Student's t-test or one-way analysis of variance (ANOVA) with Tukey's multiple comparison test was used to compare between treatment groups. Tumor growth curves were analysed using two-way ANOVA with Tukey's multiple comparison test. Survival differences between treatment groups in vivo were determined using log-rank (Mantel-Cox) analysis.

For manuscripts utilizing custom algorithms or software that are central to the research but not yet described in published literature, software must be made available to editors and reviewers. We strongly encourage code deposition in a community repository (e.g. GitHub). See the Nature Research guidelines for submitting code & software for further information.

Data

Policy information about availability of data

All manuscripts must include a data availability statement. This statement should provide the following information, where applicable:

- Accession codes, unique identifiers, or web links for publicly available datasets
- A list of figures that have associated raw data
- A description of any restrictions on data availability

Transcriptomic data from this study have been deposited in the Sequence Read Archive and can be assessed via this link: https://www.ncbi.nlm.nih.gov/sra/PRJNA864115
Accession code: PRJNA864115

Field-specific reporting

Please select the one below that is the best fit for your research. If you are not sure, read the appropriate sections before making your selection.

☒ Life sciences ☐ Behavioural & social sciences ☐ Ecological, evolutionary & environmental sciences

For a reference copy of the document with all sections, see nature.com/documents/nr-reporting-summary-flat.pdf

Life sciences study design

All studies must disclose on these points even when the disclosure is negative.

Sample size	For in vivo studies, 4 to 10 mice per treatment group were sufficient to detect meaningful biological differences with good reproducibility. For in vitro experiments, we typically performed at least 3 independent experiments. We determined the sample size to be sufficient based on the lab's and our collaborator's experience and publications.
Data exclusions	No data were excluded.
Replication	All the findings in this study were reliably reproduced in multiple independent experiments. For all in vitro experiments, our data represent at least two independent assays that produce similar results.
Randomization	Animals used were randomly assigned to each treatment groups for every animal experiment.
Blinding	Blinding was not performed for our in vivo experiments as we did not have additional staff or resources available to do so during the majority of the duration the experiments were carried out.

Reporting for specific materials, systems and methods

We require information from authors about some types of materials, experimental systems and methods used in many studies. Here, indicate whether each material, system or method listed is relevant to your study. If you are not sure if a list item applies to your research, read the appropriate section before selecting a response.

Materials & experimental systems

n/a	Involved in the study
☐	☒ Antibodies
☐	☒ Eukaryotic cell lines
☒	☐ Palaeontology and archaeology
☐	☒ Animals and other organisms
☒	☐ Human research participants
☒	☐ Clinical data
☒	☐ Dual use research of concern

Methods

n/a	Involved in the study
☒	☐ ChIP-seq
☐	☒ Flow cytometry
☒	☐ MRI-based neuroimaging

Antibodies

Antibodies used

All antibodies have been described as required in methods and also listed here.
Immune checkpoint antibodies, immune cell depletion antibodies and their corresponding isotype controls for in vivo studies obtained from BioXCell: anti-mouse PD-1 mAb (clone: RMP1-14, BE0146), anti-mouse CD8 mAb (clone: YTS 169.4, BE0117), anti-mouse CD4 mAb (clone: GK1.5, BE0003-1), Rat IgG2a isotype control mAb (clone:2A3, BE0089) and Rat IgG2b isotype control mAb (clone: LTF2, BE0090).

Apoptosis analysis: APC-conjugated Annexin V (405717, Becton Dickinson)

Flow cytometry analysis of γH2AX: p-γH2AX Ser139 (9718, Cell Signalling Technology), Anti-rabbit IgG (H+L), F(ab')2 Fragment - Alexa Fluor® 488 Conjugate (4408, Cell Signaling Technology).

Immunofluorescence analysis of γ-H2AX: histone γ-H2AX (Ser139) rabbit mAb (1:200 in blocking buffer; 9718, Cell Signaling Technology), Anti-rabbit IgG (H+L), F(ab')2 Fragment - Alexa Fluor® 488 Conjugate (4408, Cell Signaling Technology)

Western blot antibodies from Cell Signalling Technologies: p-CHK1 Ser345 (2348), p-TBK1 Ser172 (5483), p-IRF3 Ser396 (4947), STING (13647), p- GAPDH (5174), α-tubulin (2144), β-actin (4970), Goat anti-rabbit Ig HRP-linked secondary antibody (7074)

Western blot antibodies from Bethyl Laboratories Inc: RPA32 (A300-244A) and p-RPA32 Ser4/Ser8 (A300-245A).

Assessing MHC expression on human tumor cell surface via flow cytometry: HLA-ABC (PE, clone G46-2.6, 555553, BD Biosciences), HLA-DR (APCCy7, clone LT43, 502515, BD Biosciences)

Assessing calreticulin expression on tumor cell surface: Alexa Fluor®647-conjugated calreticulin antibody (EPR3924, ab196159, Abcam).

Analysis of mouse tumour-infiltrating immune subsets via Flow cytometry: CD45.2 (clone: 104; 1:200 dilution, 109824, Biolegend), TCRβ (1:200 or 1:400 dilution; clone: H57-597; 563221, BD Biosciences; 109241, 109226, Biolegend; 12-5961-83, 17-5961-83, 11-5961-85, 45-5961-82, eBioscience), CD4 (clone: RM4-5; 1: 400 dilution; 100551, Biolegend; clone: Gk1.5; 1:200 dilution; 25-0041-82, eBioscience), CD8a (clone: 53-6.7; 1:400 dilution; 100748, Biolegend), CD11b (clone: M1/70; 1:400 dilution; 101242, Biolegend), CD11c (clone: N418; 1:400 dilution; 117335, Biolegend; 1:200 dilution; 53-0114-82, eBioscience), CD279 (PD-1; clone: J43; 1:200 dilution; 11-9985-85, eBioscience), FOXP3 (clone: FJK-16s; 1: 200 dilution; 48-5773-82, eBioscience), Granzyme B (clone: GB11; 1:200 dilution; 515406, BioLegend; clone: NGZB; 1:100 dilution; 25-8898-82, eBioscience), Ki67 (clone: 16A8; 1:200 dilution; 652408, Biolegend), IFNγ (clone: XMG1.2; 1:200 dilution; 12-7311-82, eBioscience; 505839, Biolegend), CD83 (clone: Michael-19; 1:200 dilution; 121515, 1215158, Biolegend), CD103 (clone: 2E7, 1:200 dilution; 46-1031-82, 1:400 dilution; 12-1031-83, eBioscience), CD274 (B7-H1/PD-L1, clone: MIH5, 1:200 dilution; 25-5982-82, 1:400 dilution: 12-5982-82, eBioscience), MHCII (1A/1E, clone: M5/114.15.2; 1:400 dilution; 17-5321-82; eBioscience, 1:200 dilution; 107620, Biolegend), CD80 (clone: 16-10A1, 1:400 dilution; 104729, Biolegend), CD86 (clone Gl1, 1:400 dilution; 105028, 105037, Biolegend), MHCI (H2Kb; clone: AF6-88.5; 1:400 dilution; 116520, Biolegend), MHCI (H2Db, clone: 28-14-8, 1:200 dilution; 48-5999-82 eBioscience), MHCI (H2Kb/H2Db, clone: 28.8.6, 1:400 dilution; 114612, Biolegend).

Validation

All antibodies used in our study have been validated by the manufacturers and detailed information can be found on the manufacturers' websites via these links:

Cell Signalling Technologies: https://www.cellsignal.com/
Validation statement for Cell Signalling Technolgies: https://www.cellsignal.com/about-us/cst-antibody-validation-principles

BioXCell: https://bxcell.com/

Bethyl Laboratories: https://www.fortislife.com/bethyl-laboratories
Validation statement for Bethyl Laboratories: https://www.fortislife.com/antibody-validation

eBioscience: https://www.thermofisher.com/au/en/home/life-science/antibodies/ebioscience.html

Biolegend: https://www.biolegend.com/
Quality control for Biolegend: https://www.biolegend.com/en-us/quality/quality-control

BD Biosciences: https://www.bdbiosciences.com/en-us

Abcam: https://www.abcam.com/
Validation statement from Abcam: https://www.abcam.com/primary-antibodies/how-we-validate-our-antibodies

Eukaryotic cell lines

Policy information about cell lines

Cell line source(s)	MDA-MB-231, MDA-MB-468. MDA-MB-453, MDA-MD-436, HCC1806 were obtained via ATCC. The AT3 and AT3OVA mouse TNBC cell lines were obtained from our collaborator and co-author of this study Prof Phil Darcy. The 4T1ch9 TNBC cell line was obtained from our collaborator Prof Robin Anderson (the Olivia Newton John Cancer Research Institution, Victoria, Australia).
Authentication	All the human cell lines were authenticated via short tandem repeat analysis. The mouse cell lines were not authenticated
Mycoplasma contamination	All cell lines were routinely tested for mycoplasma contamination and we confirm that all cell lines were negative for mycoplasma contamination.
Commonly misidentified lines (See ICLAC register)	No commonly misidentified lines were used in this study.

Animals and other organisms

Policy information about studies involving animals; ARRIVE guidelines recommended for reporting animal research

Laboratory animals	This study used female mice in our animal studies. The mice were 6-8 weeks old at the start of the experiments. Strains used: C57Bl/6, C57BL/6 RAG1-/-, C57BL/6 RAG2-/-γc-/- and BALB/c.
Wild animals	No wild animals were used in this study
Field-collected samples	No field collected studies were used in this study
Ethics oversight	All animal experiments were approved by the Peter MacCallum Cancer Centre Animal Experimentation Ethics Committee (E556 and E628) and conducted in accordance with the National Health and Medical Research Council Australian Code of Practice for the Care and Use of Animals for Scientific Purposes.

Note that full information on the approval of the study protocol must also be provided in the manuscript.

Flow Cytometry

Plots

Confirm that:

☒ The axis labels state the marker and fluorochrome used (e.g. CD4-FITC).

☒ The axis scales are clearly visible. Include numbers along axes only for bottom left plot of group (a 'group' is an analysis of identical markers).

☒ All plots are contour plots with outliers or pseudocolor plots.

☒ A numerical value for number of cells or percentage (with statistics) is provided.

Methodology

Sample preparation

Tumor collection and preparation for flow cytometry analysis of tumor-infiltrating immune subsets
The mice are euthanized via cervical dislocation. Tumours were excised and digested using a mix of 1mg/ml collagenase type IV (Sigma Aldrich) and 0.02mg/ml DNAase (Sigma Aldrich). After 30 minutes of digestion at 37 ºC, cells were passed through a 70 µm filter twice. For analysis of intracellular IFNγ, cells were stimulated with 50 ng/ml phorbol 12-myristate 13-acetate (PMA; Sigma Aldrich), 1 µg/ml Ionomycin (Sigma Aldrich), GolgiSTOP (1:1500 dilution; Becton Dickinson) and GolgiPLUG (1:1000 dilution; Becton Dickinson) for 4 hours at 37ºC. Single cell suspensions were then stained with the appropriate antibodies and analysed by flow cytometry analysis on LSR II, BD LSRFortessa™ or FACSymphony™ flow cytometers (BD Biosciences).

Apoptosis analysis
Cells were plated in 24 well plates and treated the following day with the indicated agents for 72 hours. Cells were then resuspended in 100 µL 1x annexin V binding buffer (5x: 50mM HEPES, 700mM NaCl, 12.5mM CaCl2, pH7.4) and stained with 1 µg/ml propidium iodide and APC-conjugated Annexin V (1:100 dilution, 405717, Becton Dickinson). Cells were analysed using BD Biosciences LSR II flow cytometer. All experiments were performed in triplicate with three independent experiments. The gating strategy is provided in Supplementary Figure 8a.

Flow cytometry analysis of γH2AX
Cells were seeded in 24 well plates and treated the following day with the indicated agents. Cells were stained with a viability marker (LIVE/DEAD™ Fixable Yellow Dead cell stain kit, ThermoFisher Scientific) before resuspended in 4% paraformaldehyde (Sigma Aldrich). Cells were permeabilised by resuspending in 90% methanol. Cells were then stained with p-γH2AX Ser139

(1:50 dilution. 9718, Cell Signalling Technology) overnight at 4ºC. The next day, cells were incubated with fluorochrome-conjugated secondary antibody Anti-rabbit IgG (H+L), F(ab')2 Fragment - Alexa Fluor® 488 Conjugate (1:200 dilution, 4408, Cell Signalling Technology). Flow cytometry analysis was performed on an LSR II flow cytometer (BD Biosciences). FACS data was analysed using FlowJo version 10 software (Tree Star Inc., USA). All experiments were performed in triplicate with at least three independent experiments. The gating strategy is provided in Supplementary Figure 8b.

Assessing MHC expression on tumor cell surface
Cells were seeded in 24-well plates and treated the next day with indicated treatments. After 72h, the adherent cells were lifted using Tryple (Gibco) and stained with HLA-ABC (clone G46-2.6, 1:200 dilution, 555553, BD Biosciences), HLA-DR (clone LT43, 1:200 dilution, 502515, BD Biosciences) and a viability marker (1:400 dilution, LIVE/DEAD™ Fixable Yellow Dead cell stain kit, ThermoFisher Scientific) before resuspended in 4% paraformaldehyde (Sigma Aldrich). Cells were analysed using the BD LSR II cell analyser. FACS data was analysed using FlowJo version 10 software (Tree Star Inc., USA). All experiments were performed in triplicate with three independent experiments. The gating strategy is provided in Supplementary Figure 9a.

Assessing calreticulin expression on tumor cell surface
Cells were seeded in 24-well plates and treated the next day with indicated treatments. After 72h, cells in suspension and adherent cells were collected and centrifuged at 1400 rpm at 4oC for 4 min. Cells were washed once with PBS and stained with Alexa Fluor®647-conjugated calreticulin antibody (1:50 dilution, clone: EPR3924, ab196159 Abcam). After 30 min incubation on ice, cells were washed with PBS and resuspended in 10 µg/mL propidium iodide. After incubation for 30 min at room temperature, cells were analysed using the BD LSR II cell analyser. FACS data was analysed using FlowJo version 10 software (Tree Star Inc., USA). All experiments were performed in triplicate with three independent experiments. The gating strategy is provided in Supplementary Figure 9b.

Instrument	Flow cytometric data were collected using BD Biosciences LSRII, BD LSRFortessa and BD FACSymphony cell analysers.
Software	FACS data was analysed using FlowJo version 10 software (Tree Star Inc., USA).
Cell population abundance	Flow sorting was not used in this study.
Gating strategy	FSC SSC to remove debris; FSC-H by FSC-A to define single cells. The gating strategy used for all relevant experiments were shown in Supplementary Figures 9, 10, 12, 13.

☒ Tick this box to confirm that a figure exemplifying the gating strategy is provided in the Supplementary Information.
